# A hybrid fuzzy-PIDD² control strategy for coordinated LFC and AVR in renewable-integrated multi-area power systems

**DOI:** 10.1038/s41598-025-34481-7

**Published:** 2026-01-27

**Authors:** Mohamed H. T. Omar, Ragi A. Hamdy, Hossam Kotb

**Affiliations:** Department of Electrical Engineering , Alexandria University , Alexandria, 21544 Egypt

**Keywords:** Fuzzy control, Load frequency control, Optimization, Automatic voltage regulation, Renewable energy, Two area interconnected power system, Energy science and technology, Engineering, Mathematics and computing

## Abstract

This paper presents a novel hybrid control strategy that integrates a Fuzzy Proportional–Integral–Derivative Double Derivative (FPIDD²) controller with a conventional PIDD² controller for Load Frequency Control (LFC) and Automatic Voltage Regulation (AVR) in multi-area interconnected power systems, respectively. The proposed FPIDD²+PIDD² hybrid scheme enhances the overall dynamic stability and robustness of power systems operating under high renewable energy penetration. Controller parameters are optimally tuned using different metaheuristic algorithms, namely the Particle Swarm Optimization (PSO), Gorilla Troops Optimizer (GTO), and Marine Predators Algorithm (MPA). The proposed hybrid controller’s performance is evaluated against conventional PID and standalone PIDD² controllers under various disturbances, including step load changes, random load variations, and renewable energy fluctuations, where control performance is evaluated based on the Integral of Time-weighted Absolute Error (ITAE) criterion. Within the simulated test cases, the proposed FPIDD²+PIDD² controller achieves notable performance improvement, reducing ITAE by up to 94% and 90% compared to conventional PID and standalone PIDD² controllers, respectively. These results confirm the hybrid controller’s smooth transient response, enhanced damping, and improved robustness against nonlinearities and system uncertainties.

## Introduction

### Background and research gap

Modern power systems have become increasingly complex due to the global surge in electricity demand and the extensive integration of renewable energy sources (RES) such as wind and solar power^1^. The intermittent and uncertain nature of these resources, combined with unpredictable load variations, generator outages, and nonlinear interactions between interconnected areas, makes system stability and reliability more difficult to maintain^2–4^. These fluctuations lead to deviations in both frequency and voltage, which can degrade efficiency, damage sensitive equipment, and even trigger large-scale cascading blackouts^5–7^. Traditional control frameworks employing Load Frequency Control (LFC) and Automatic Voltage Regulation (AVR) schemes, which are typically based on conventional PID controllers, have long been used to stabilize power systems^8^. However, PID controllers are generally tuned for nominal operating points and exhibit degraded performance under nonlinear, time-varying, and highly coupled conditions^9,10^. Their inability to dynamically adapt to the changing dynamics of systems with high renewable penetration, electric vehicle (EV) charging variability, and random load disturbances limit their effectiveness in ensuring overall system robustness. While recent years have witnessed progress in intelligent and optimization-based controllers, there remains a lack of a unified, adaptive, and optimally tuned control strategy that simultaneously manages LFC, AVR, and tie-line power deviations under complex real-world conditions involving nonlinearities, RES integration, EVs, and stochastic load variations^11–14^. Therefore, addressing this specific gap is crucial for improving the resilience and reliability of future power systems, ensuring stable operation despite uncertain and rapidly changing grid environments^15–17^.

### Literature review

Modern power systems have witnessed increasing complexity due to the growing penetration of renewable energy and the need for robust control mechanisms. In this context, several enhanced control strategies have been proposed to improve system dynamic performance, stability, and resilience under varying operational conditions. Gu et al.^18^ presented a comprehensive survey of modern control architectures for resilient power grids, emphasizing the integration of adaptive, predictive, and intelligent controllers to ensure operational reliability under disturbances. Building on this foundation, Sahu et al.^19^ developed and analyzed a Proportional–Integral–Derivative Double Derivative (PIDD²) controller for load frequency control (LFC) in deregulated environments, demonstrating superior damping and faster convergence compared with classical PID controllers. Ray and Paital^20^ further improved the robustness of the PIDD² approach for hybrid power systems, achieving better frequency regulation under renewable variability. Dash et al.^21^ investigated the impact of advanced thyristor-controlled series capacitors in dual-area LFC–AVR systems using interval type-2 fuzzy PID structures, confirming the advantages of higher-order control approaches under nonlinear conditions. Recent advancements have also focused on fractional-order and hybrid PID-based control. Gupta^22^ introduced a fractional-order PID controller for LFC in deregulated hybrid systems, reporting significant robustness improvements, while Wang et al.^23^ applied fractional-order control in systems integrated with hydrogen energy storage. Alnefaie et al.^24^ further optimized multi-area LFC–AVR performance through advanced cascaded controllers under renewable uncertainty.

In parallel, fuzzy logic control has been recognized as a powerful alternative to conventional methods. Zadeh^25^ originally formulated the concept of fuzzy logic for control systems, establishing the basis for reasoning under uncertainty. Building on this principle, Mishra and Das^26^ applied an adaptive fuzzy logic–based LFC scheme in renewable-integrated power systems and demonstrated its superior robustness under parameter variations. Hannan et al.^27^ provided a critical review of fuzzy logic applications for smart grids, confirming its effectiveness in handling nonlinearities and model uncertainties. A. Ali, G. Biru and Bantyirga^28^ examined the design and potential application of fuzzy logic-based AGC and AVR schemes for multi-area systems, validating their capability in improving voltage and frequency stability. Khan et al.^29^ further extended fuzzy logic concepts to vehicle-to-grid applications, integrating an adaptive fuzzy AVR controller for ancillary services support in smart grid environments. More recently, Mansour et al.^30^ enhanced combined LFC–AVR operation using a fuzzy PID controller, and Kalyan et al.^31^ proposed a hybrid fuzzy PID approach optimized via the HAEFA algorithm to improve coordinated control performance.

Optimization-based controller tuning has also gained widespread attention in the literature. Gad^32^ offered a systematic review of Particle Swarm Optimization (PSO) and its applications, emphasizing its simplicity and efficiency in handling multidimensional search spaces. Houssein et al.^33^ discussed major advances in PSO theory and application, focusing on convergence improvements. Fang et al.^34^ provided a recent survey highlighting algorithmic trends and hybridization techniques for PSO. Sengupta et al.^35^ and Grassi et al.^36^ introduced hybridized PSO frameworks that enhance exploration and exploitation balance, while Sienz and Innocente^37^ demonstrated PSO’s application in engineering scheduling problems, confirming its suitability for practical optimization tasks in control systems. Izci et al.^38^ recently optimized advanced PID controllers using hybrid metaheuristics for dynamic load frequency control, while Doan and Nguyen^39^ proposed a multi-stage hybrid smart controller considering renewable and HVDC integration.

Beyond PSO, the Marine Predator Algorithm (MPA) and Gorilla Troops Optimizer (GTO) have emerged as effective metaheuristics for complex optimization challenges. Faramarzi et al.^40^ first introduced the MPA inspired by predator-prey dynamics, offering fast convergence and strong global search capability. Singh et al.^41^ applied the MPA to engineering design optimization, validating its efficiency. Su et al.^42^ enhanced the algorithm through Lévy flight and Brownian motion mechanisms, while Jang et al.^43^ provided a detailed review of MPA variants and engineering applications. Xie et al.^44^ and Zadeh et al.^45^ evaluated MPA performance against other global optimizers, highlighting its adaptability, and Zhang et al.^46^ analyzed the impact of environmental factors on MPA’s convergence characteristics. Similarly, Mirjalili and Kazem^47^ proposed the Gorilla Troops Optimizer (GTO), a social-behavior–inspired metaheuristic demonstrating strong exploration properties. Fard and Wang^48^ combined GTO with genetic algorithms to improve multi-objective optimization performance, and Chen et al.^49^ applied GTO for solving multimodal engineering problems. Zhang et al.^50^ improved the exploration–exploitation balance within GTO, while Wang et al.^51^ analyzed its performance in real-world engineering design problems. Araujo and Andrade^52^ hybridized GTO with PSO to address multi-objective scenarios effectively, and Yadav and Bhavsar^53^ conducted a comparative study confirming its competitiveness against other evolutionary algorithms. Mishra et al.^54^ applied adaptive optimization techniques for LFC in multi-area power systems with energy storage, illustrating the practical relevance of these algorithms. Wang and Li^55^ further showed that coordinated LFC–AVR control, optimized through such algorithms, enhances overall system dynamic performance under renewable integration. Complementary reviews such as Raja Sathish Kumar et al.^56^ have emphasized the importance of smart control strategies in achieving these improvements.

Because power systems are inherently complex and geographically distributed, accurate yet tractable modeling techniques are essential. Zhang et al.^57^ discussed challenges associated with managing the growing complexity of modern grids and proposed methods for reducing model order without compromising essential dynamics. Gupta and Chakrabarti^58^ compared different modeling techniques for stability analysis, highlighting the trade-off between computational efficiency and dynamic accuracy. Alam and Saha^59^ validated two-area interconnected power system models for renewable integration studies, confirming their accuracy in replicating real-world conditions. Wang et al.^60^ proposed simplified dynamic models to facilitate stability and control analysis, while Pandey and Bansal^61^ introduced reduced-order modeling approaches for large-scale power systems. To represent the interconnection characteristics between regions, Chen et al.^62^ studied two-area to multi-area system dynamics, showing how tie-line interactions affect overall stability. Fernandez et al.^63^ conducted a detailed two-area case study on power flow coordination, and Li and Mi^64^ identified frequency regulation challenges associated with inter-area coupling. Kundur and Morison^65^ contributed foundational insights into the dynamic behavior of interconnected systems, establishing the two-area model as a benchmark for theoretical and experimental studies. Recent works have continued refining these modeling approaches. Bevrani and Hiyama^66^ emphasized balancing modeling accuracy and computational simplicity in power system simulations. Sharma et al.^67^ proposed computationally efficient dynamic models for real-time studies, and Eltamaly et al.^68^ extended these frameworks from two-area to large-scale configurations. Recent review studies such as Sharma and Patel^69^, and Doan and Nguyen^70^ have further highlighted the growing need for intelligent and hybrid control strategies to address LFC–AVR coordination challenges in renewable-rich environments. Based on the insights from these studies, the two-area interconnected power system model is adopted in this work as a practical yet accurate representation of large-scale systems. This model facilitates the evaluation of the proposed control strategy while maintaining computational efficiency and ensuring dynamic fidelity.

### Paper organization and contribution

The paper begins with system modeling shown in Sect. 2, followed by the proposed controllers’ structures in Sect. 3, while Sect. 4 provides performance evaluations through simulation results and comparative analyses conducted across four case studies. The study concludes in Sect. 5 with key findings, practical insights, and future research directions, ensuring a logical and reproducible flow throughout. The main contributions of this study could be summarized as the following:

- Introducing a hybrid FPIDD² controller, integrating fuzzy logic with the PIDD² structure to effectively enhance the two-area interconnected power systems dynamic stability.
- Demonstrating the adaptability of controllers, including PID, PIDD², and FPIDD²+PIDD² across diverse scenarios, including various load disturbances, high renewable penetration, and grid conditions.
- Conducting a detailed comparison between the proposed control scheme (FPIDD²+PIDD²) and the traditional PID as well as standard PIDD² controllers, using transient response metrics and ITAE as the fitness function.
- Employing diverse metaheuristic algorithms, including PSO, MPA, and GTO for controller tuning, and evaluates their influence on performance and consistency.

## Investigated system

### System description

**Fig. 1:**
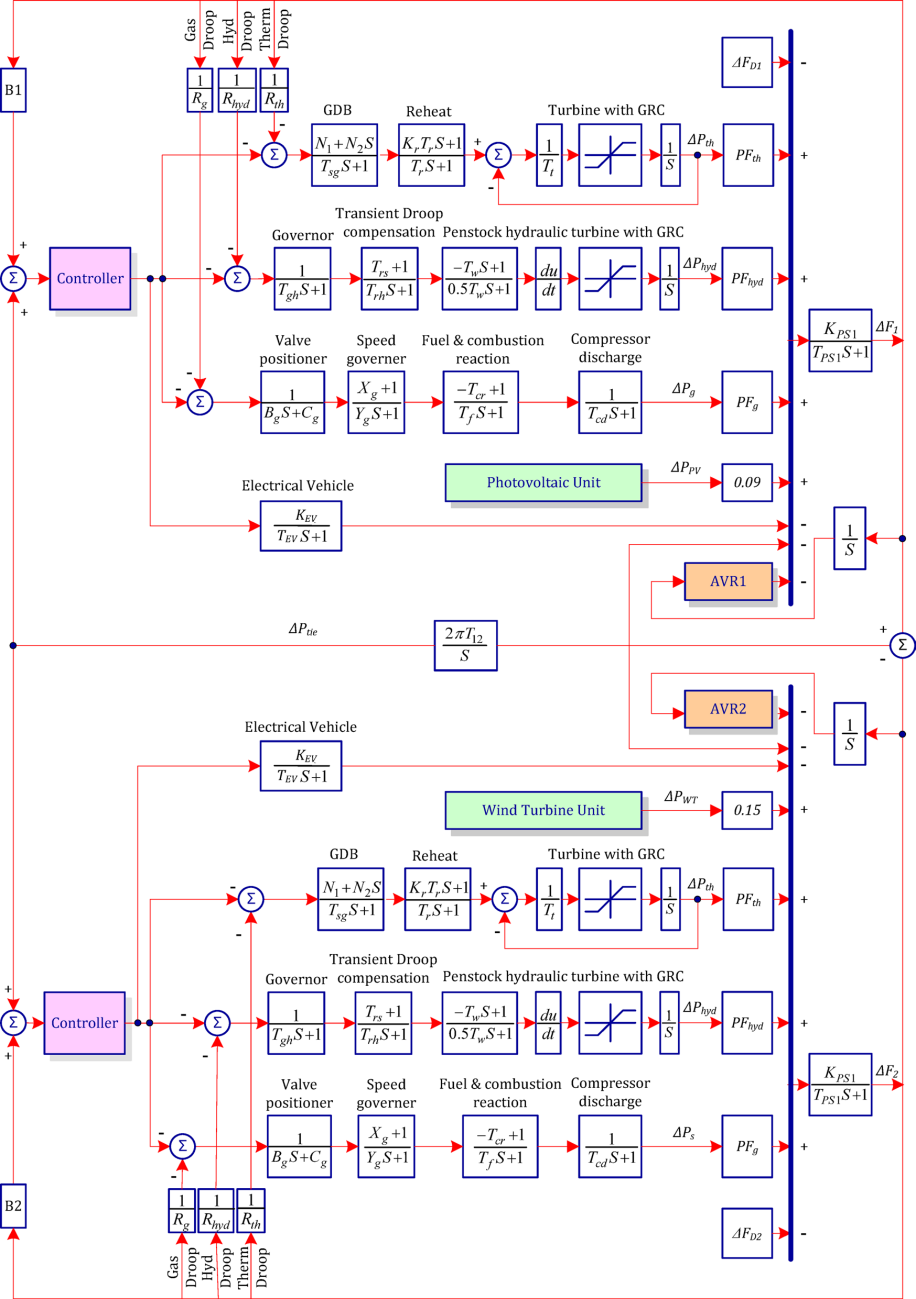
Block diagram of the investigated two-area interconnected power system.

As shown per Fig. 1, the study investigates a two-area interconnected power system, in which each area contains conventional generation units with a nominal load of 1740 MW and a capacity of 2000 MW. The generation mix includes thermal (1000 MW), gas (240 MW), and hydro (500 MW) plants. Furthermore, the system incorporates renewable energy sources (RES), with a 420 MW wind power unit connected to Area-2 and a 300 MW photovoltaic (PV) unit injected in Area-1, producing a total of 720 MW^71^. Each area also includes electric vehicle (EV) charging units, adding complexity to system dynamics. Detailed system configuration and parameters are presented in Table 1. The study illustrates the following two key nonlinearities that affect system performance:

- Generation Rate Constraint (GRC), which is driven by physical limitations of thermal and hydro units, restricts how fast generators can change output, introducing nonlinear dynamics.
- Governor Dead Band (GDB), which creates a non-responsive zone where minor input changes do not affect governor output, reducing control sensitivity during small disturbances^72^.

For hydropower, the GRC is 360% p.u./min (0.06 p.u. MW/s) for decreasing and 270% p.u./min (0.045 p.u. MW/s) for increasing output; for thermal units, it is 10% p.u./min (0.0017 p.u. MW/s) in both directions^73^. Accurate modeling of these effects is essential for reliable simulation and control design^74,75^.

**Table 1:** Power system parameters.

Model Constant	Initial Magnitude	Model Constant	Initial Magnitude	Model Constant	Initial Magnitude
*B*_*g*_	0.0490 s	*PF*_*Th*_	0.5747	*T*_*n*_	1
*C*_*g*_	1	*PF*_*WT*_, *PF*_*PV*_	0.025	*T*_*ps1*_, *T*_*ps2*_	11.49
*K*_*a*_	10	*R*_*Th*_, *R*_*hyd*_, *R*_*g*_	2.4 Hz/MW	*T*_*r*_	10.2000 s
*K*_*e*_, *K*_*n*_, *K*_*s*_	1	*T*_*12*_	0.0433 MW	*T*_*rh*_	28.7490 s
*K*_*EV*_	1	*T*_*a*_	0.1	*T*_*rs*_	4.9000 s
*K*_*ps1*_, *K*_*ps2*_	68.96	*T*_*cd*_	0.2000 s	*T*_*s*_	0.01
*K*_*r*_	0.3	*T*_*cr*_	0.0100 s	*T*_*sg*_	0.0600 s
*N*_*1*_	0.8	*T*_*e*_	0.4	*T*_*t*_	0.3000 s
*N*_*2*_	− 0.2/π	*T*_*EV*_	1	*T*_*w*_	1.1000 s
*PF*_*g*_	0.138	*T*_*f*_	0.2390 s	*X*_*g*_	0.6000 s
*PF*_*hyd*_	0.287	*T*_*gh*_	0.2000 s	*Y*_*g*_	1.1000 s

### Automatic voltage regulation

**Fig. 2:**
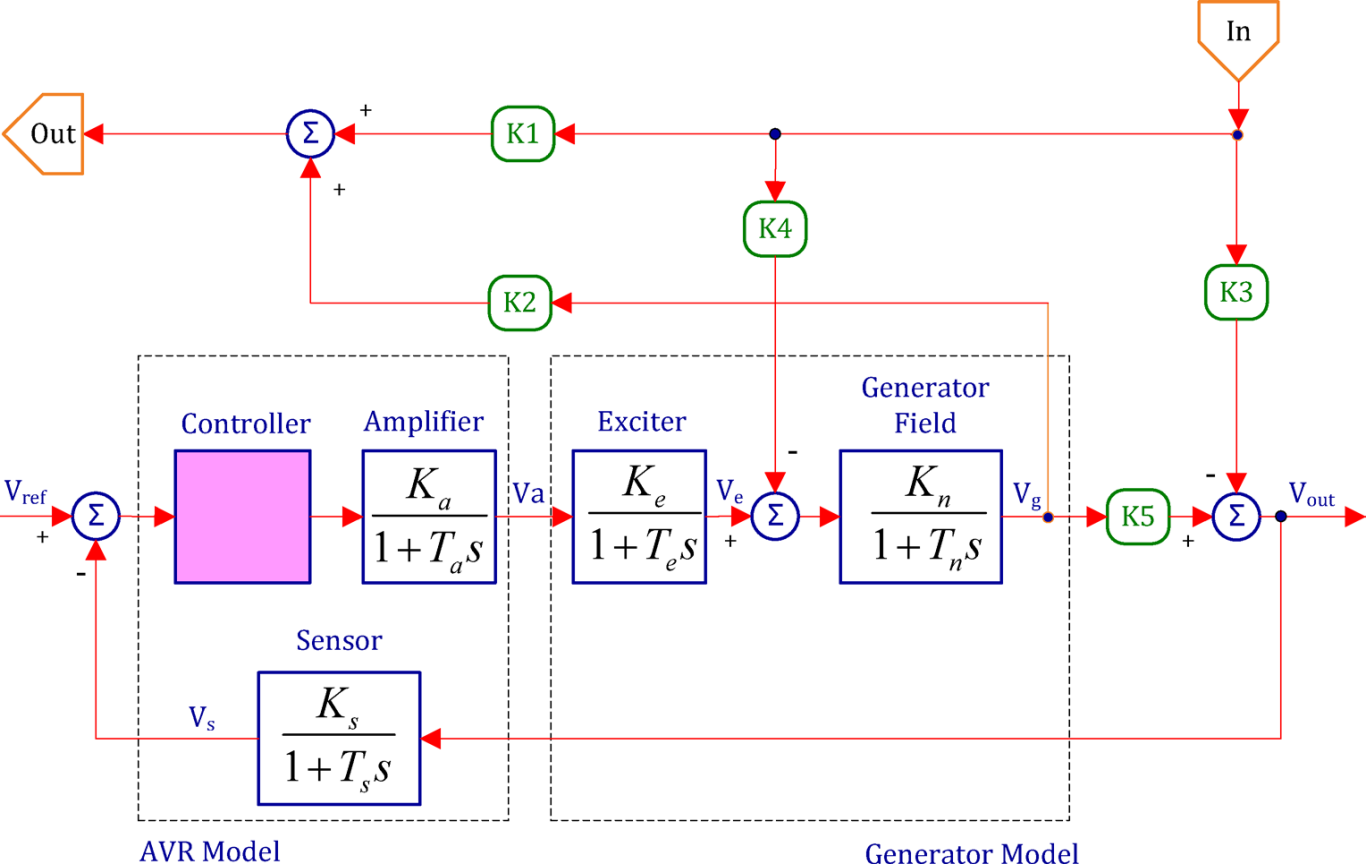
Schematic diagram of the Automatic Voltage Regulation (AVR) loop with coupling coefficients.

The generator’s terminal voltage (V_g_) can fluctuate in response to changes in reactive power load, as illustrated in Fig. 2.

As a result, the AVR system seeks to reduce reactive power losses brought on by voltage discrepancies between the target output voltage (V_out_) and the terminal voltage (V_e_) of the exciter. In this case, the generator’s terminal voltage is sensed via a single-phase potential transformer. Next, a reference voltage (V_ref_) is compared to this sensed voltage, represented by the symbol (V_s_). In order to manage the exciter’s field and, consequently, the terminal voltage, the ensuing error signal is amplified to be (V_a_). The induced electromotive force (emf) fluctuates as a result of this action, changing the field current of the generator.

The detailed coupling coefficient of the Automatic Voltage Regulation (AVR) unit for the system under study is shown per Table 2^76^.

**Table 2:** AVR coupling Coefficients.

Coupling Coefficient	Description	Value
*K1*	Represents the sensitivity of terminal voltage (V_out_) to internal EMF variations	1.5
*K2*	Reflects how small changes in stator EMF influence real power	0.3
*K3*	Denotes the impact of rotor angle changes on terminal voltage	0.1
*K4*	Demonstrates how rotor angle variances affect stator EMF	1.4
*K5*	Quantifies how variations in stator EMF affect rotor angle	0.5

### PV generation unit

**Fig. 3:**
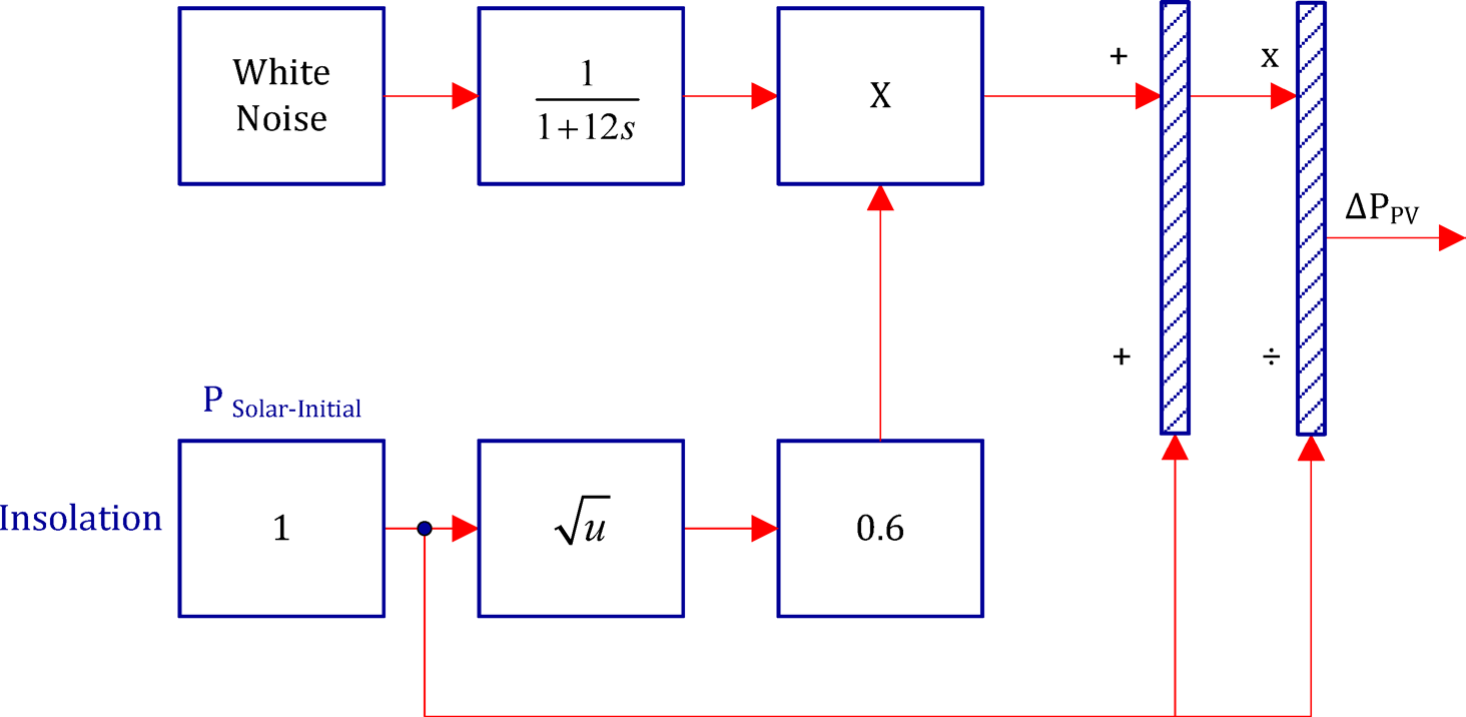
System model for PV unit^77^.

The Photovoltaic (PV) unit model in Area 1 is presented as shown in Fig. 3^77^. In this model, the white-noise block is deployed to produce random output fluctuations, which are then multiplied by the typical output power of an actual PV plant.

**Fig. 4:**
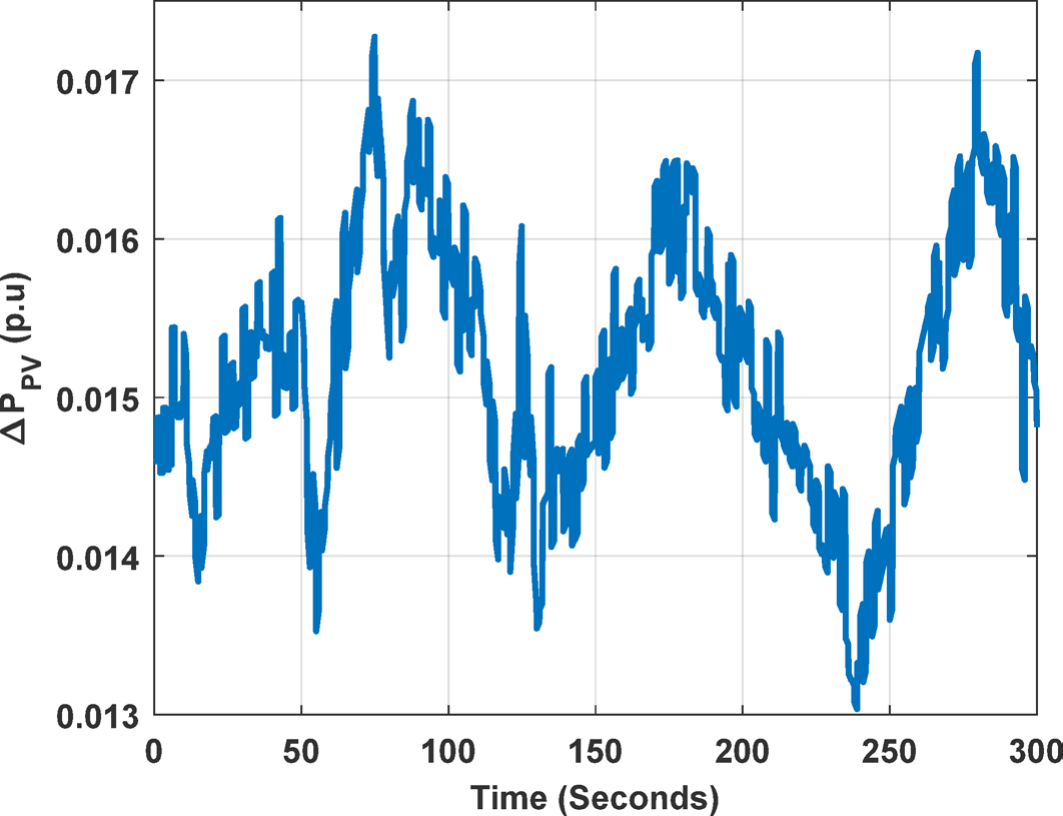
Output power of the PV unit.

The fluctuations of the output power generated by the PV plant are shown in Fig. 4.

### Wind generation unit

**Fig. 5:**
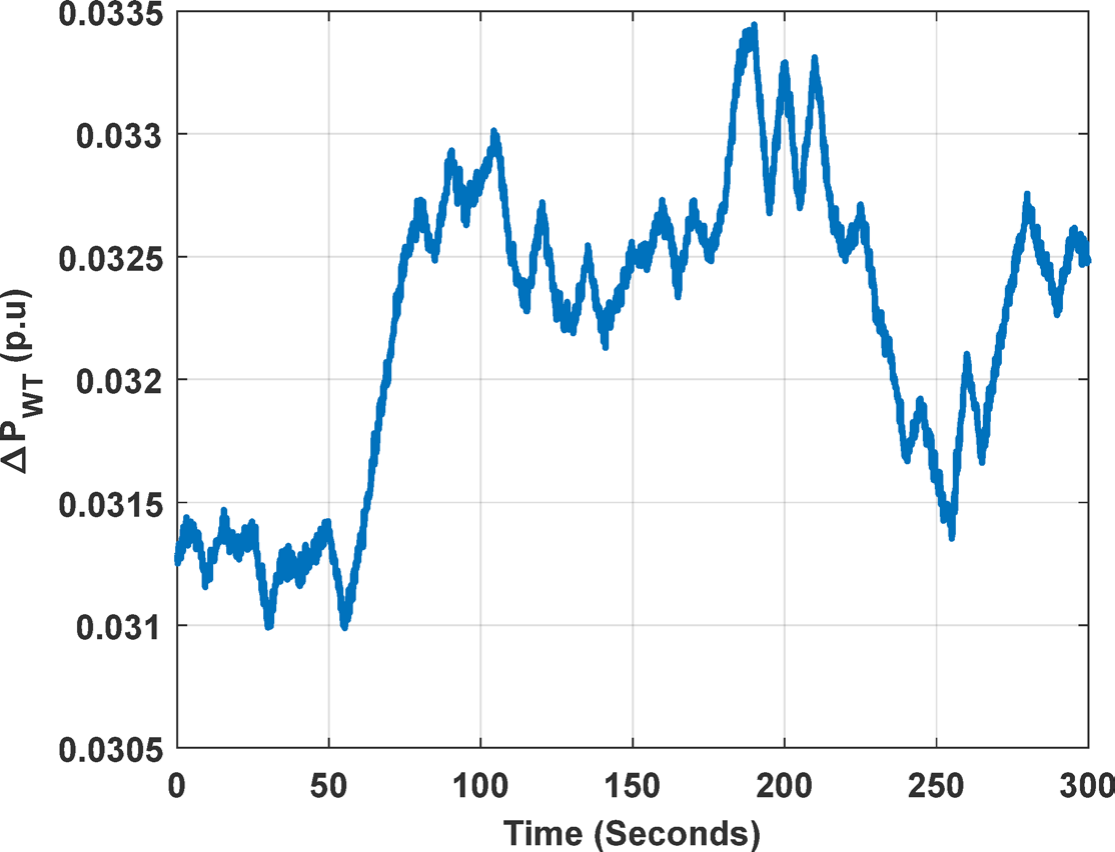
Output power of the wind-turbine unit.

**Fig. 6:**
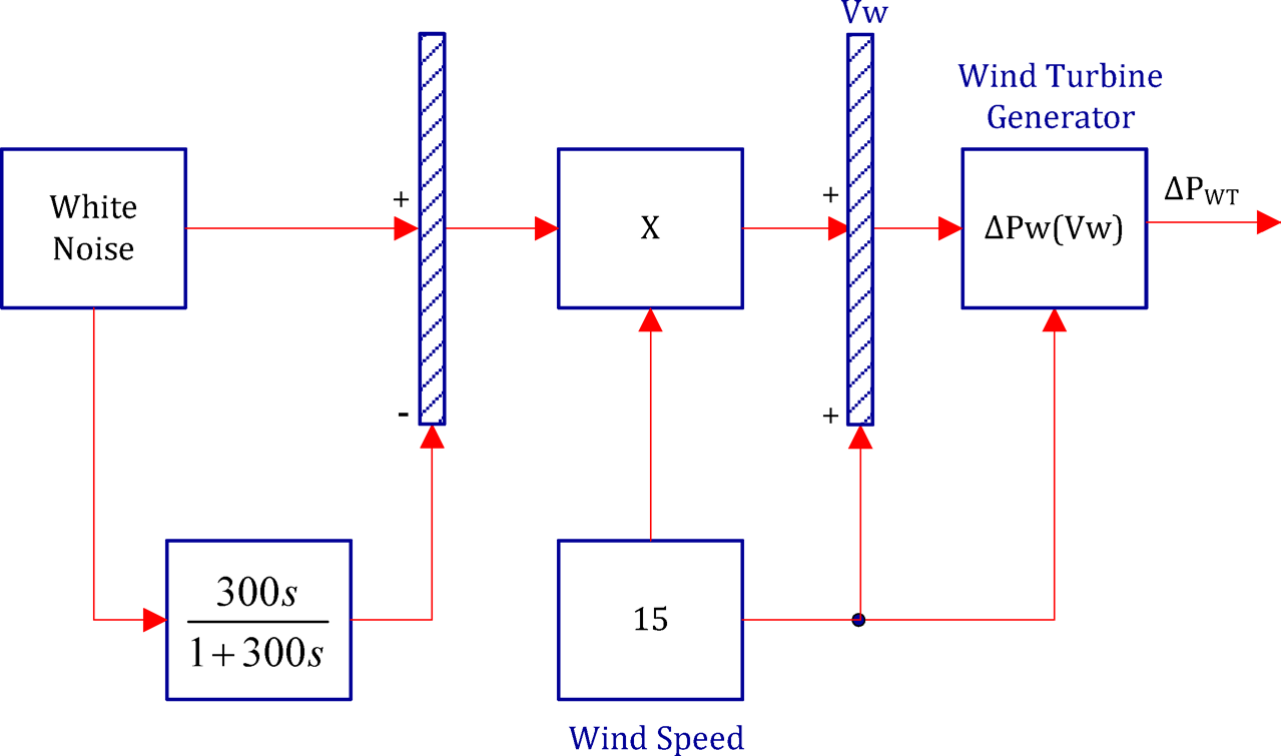
System model of the wind power plant^78^.

Figure 5 illustrates the output power of the 93 wind units, each generating 0.75 MW, resulting in a total wind farm output of approximately 70 MW. The figure represents the power trend based on actual wind farm data, and the corresponding model, shown in Fig. 6, employs a white-noise block to generate an arbitrary wind speed profile, which is multiplied by the wind speed. The wind turbine output power (Pw) is 750 kW and the rated wind speed (Vw) is 15 m/s^78^.

### Electric vehicle

Electric vehicle (EV) integration into modern power systems, especially with two-area interconnected grids, has become an important research area^79,80^. EVs are increasingly seen as not only mobile loads but also as potential distributed energy resources that can help improve grid stability^81^. When aggregated and controlled properly, EVs can support load frequency control (LFC) by acting as flexible demand or storage units, capable of discharging and/or charging according to the frequency deviations of the power system^82^. Because of this, EVs are particularly helpful in power systems that have a high penetration of renewable energy, where frequency and voltage fluctuations are more frequent^83^. In most control studies, EV dynamics are modeled using simplified linear models for easier system analysis, controller tuning, and performance evaluation^84^. This simplification is especially useful when incorporating EVs into the overall system dynamics in LFC studies, where multiple control loops, such as AVR, LFC, governor, turbine, and EV units, interact^85^. A widely used linear model for EV response is the first-order transfer function^86^. This model is favored because it captures the typical first-order lag behavior seen in load and response delays in aggregated EV systems. In addition, it provides a balance between simplicity and dynamic accuracy, enabling researchers to evaluate the interaction of EVs with conventional control elements in the system^87^. In this study, the first-order transfer function model of EV is presented as follows^88,89^:

1 $$\:{G}_{EV}\left(s\right)=\frac{{K}_{EV}}{{T}_{EV}s+1}$$

## Proposed controllers

### Controllers’ structures

As mentioned earlier, this study proposes an intelligent control strategy that employs two types of controllers. The first is a Proportional–Integral–Derivative–Double–Derivative (PIDD²) controller, implemented in the Automatic Voltage Regulation (AVR) loops. The second is a hybrid intelligent controller, referred to as the FPIDD² controller, which integrates the PIDD² structure with a Fuzzy Logic algorithm and is applied to the Load Frequency Control (LFC) loops. The detailed architectures of both the PIDD² and FPIDD² controllers are presented in the following sections.

#### PIDD^2^ controller

**Fig. 7:**
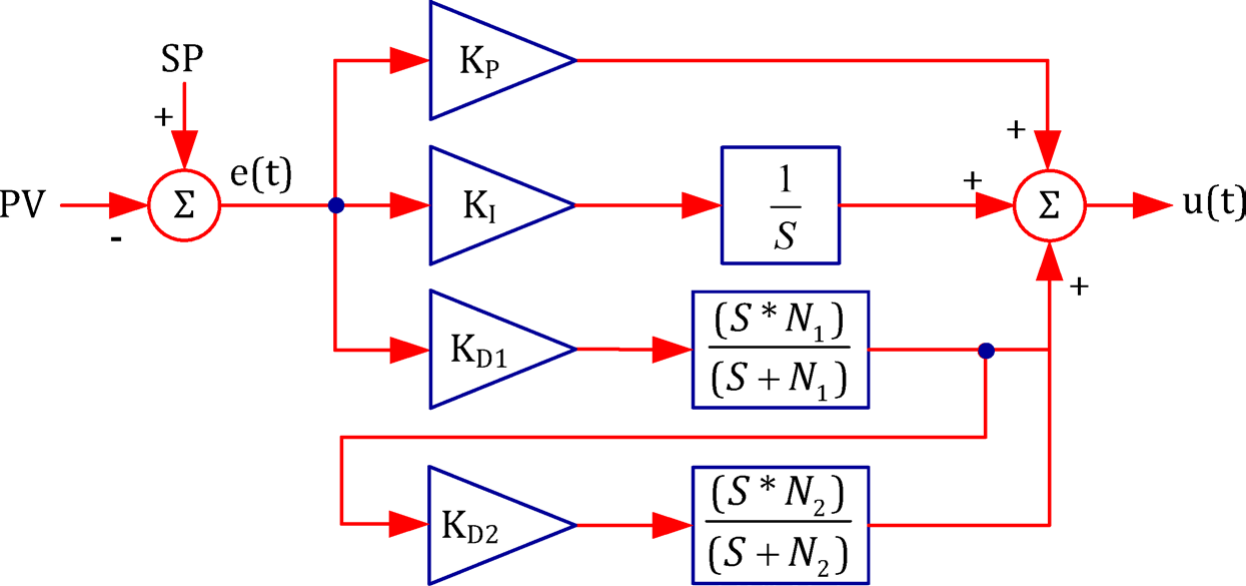
Block diagram of the PIDD² controller.

The layout of the PIDD^2^ controller is shown in Fig. 7.

The mathematical formulation of the PIDD² controller is expressed as follows^90,91^:

2 $$\:{\left.CO\left(s\right)\right|}_{PID{D}^{2}}={K}_{P}+\frac{{K}_{I}}{s}+\left[{K}_{D1}s\left(\frac{{N}_{1}}{s+{N}_{1}}\right)\right]+\left[{K}_{D1}{K}_{D2}{s}^{2}\left(\frac{{N}_{1}\:{N}_{2}}{\left(s+{N}_{1}\right)\:\left(s+{N}_{2}\right)}\right)\right]$$

The corresponding time-domain representation is:

3 $$\:{\left.CO\left(t\right)\right|}_{PID{D}^{2}}={K}_{P}\:e\left(t\right)+{K}_{i}{\int\:}_{0}^{t}e\left(\tau\:\right)d\tau\:+{K}_{d1}\:\frac{de\left(t\right)}{dt}+{K}_{d1}\:{K}_{d2}\:\frac{{d}^{2}e\left(t\right)}{d{t}^{2}}$$

#### Fuzzy logic controller

The fuzzy logic controller produces an output $\:{\left.CO\left(t\right)\right|}_{Fuzzy}$ based on the centroid (centre-of-gravity) defuzzification method, expressed as follows^92,93^:

4 $$\left. {CO(t)} \right|_{{{\mathrm{Fuzzy}}}} = \frac{{\int_{\Omega } {\mu _{o} (x) \cdot xdx} }}{{\int_{\Omega } {\mu _{o} (x)dx} }}$$

Where $\:{\mu\:}_{O}\left(x\right)$ is the aggregated output membership function of the fuzzy inference system and Ω is the universe of discourse of the output variable.

#### Fuzzy proportional integral derivative double derivative (FPIDD^2^) controller

The FPIDD² controller integrates the fuzzy inference mechanism with the PIDD² control structure. The general expression for the combined Fuzzy–PIDD² (FPIDD²) controller is given by:

5 $$\:{\left.CO\left(t\right)\right|}_{FPID{D}^{2}}=\:{\left.CO\left(t\right)\right|}_{Fuzzy}*{\left.CO\left(t\right)\right|}_{PID{D}^{2}}$$

Substituting the time-domain formulation of the PIDD² controller yields the resulting FPIDD² formula, as follows:

6 $$\:{\left.CO\left(t\right)\right|}_{FPID{D}^{2}}={\left.CO\left(t\right)\right|}_{Fuzzy}*\left[{K}_{P}\:e\left(t\right)+{K}_{i}\:{\int\:}_{0}^{t}e\left(\tau\:\right)d\tau\:+{K}_{d1}\:\frac{de\left(t\right)}{dt}+{K}_{d1}\:{K}_{d2}\:\frac{{d}^{2}e\left(t\right)}{d{t}^{2}}\right]$$

**Fig. 8:**
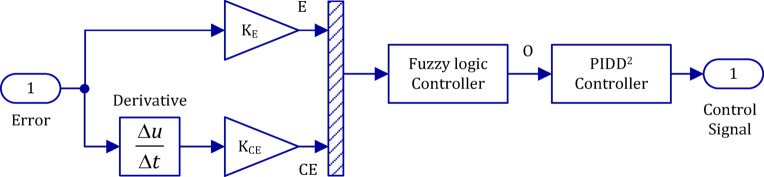
Architecture of the proposed Fuzzy PIDD² (FPIDD²) controller.

Figure 8 illustrates the structure of the FPIDD² controller, which receives two input signals, including error (E) and rate of error change (CE). The gain constants (K_E_) and (K_CE_) represent the scaling factors of the input signals, respectively^92,93^.

The two input signals Error (E) and Rate of Change of Error CE) are processed by the fuzzy logic controller, which then converts them into five linguistic variables: Large Negative (LN), Small Negative (SN), Zero (Z), Small Positive (SP), and Large Positive (LP). The number of linguistic terms in a fuzzy logic controller reflects a trade-off between control resolution and computational complexity^94^. Although three-term configurations are often adopted for simplicity, designs with seven or more terms can offer finer control precision^95^. In this study, five fuzzy sets (NL, NS, Z, PS, PL) were employed, representing a balanced choice widely reported in the literature as an effective compromise between performance and computational cost^96^. However, future work may be required to investigate alternative configurations with a higher number of linguistic terms to assess potential improvements in control accuracy^97^. Table 3 displays the Fuzzy Logic Controller’s rule basis, on which the Fuzzy Inference System (FIS) is based on Mamdani.

**Table 3:** The fuzzy logic controller’s rule basis.

Error (E)	Rate of Change of Error (CE)				
	LN (Large Negative)	SN (Small Negative)	Z (Zero)	SP (Small Positive)	LP (Large Positive)
LN (Large Negative)	LN (Large Negative)	LN (Large Negative)	SN (Small Negative)	SN (Small Negative)	Z (Zero)
SN (Small Negative)	LN (Large Negative)	SN (Small Negative)	SN (Small Negative)	Z (Zero)	SP (Small Positive)
Z (Zero)	SN (Small Negative)	SN (Small Negative)	Z (Zero)	SP (Small Positive)	SP (Small Positive)
SP (Small Positive)	SN (Small Negative)	Z (Zero)	SP (Small Positive)	SP (Small Positive)	LP (Large Positive)
LP (Large Positive)	Z (Zero)	SP (Small Positive)	SP (Small Positive)	LP (Large Positive)	LP (Large Positive)

**Fig. 9:**
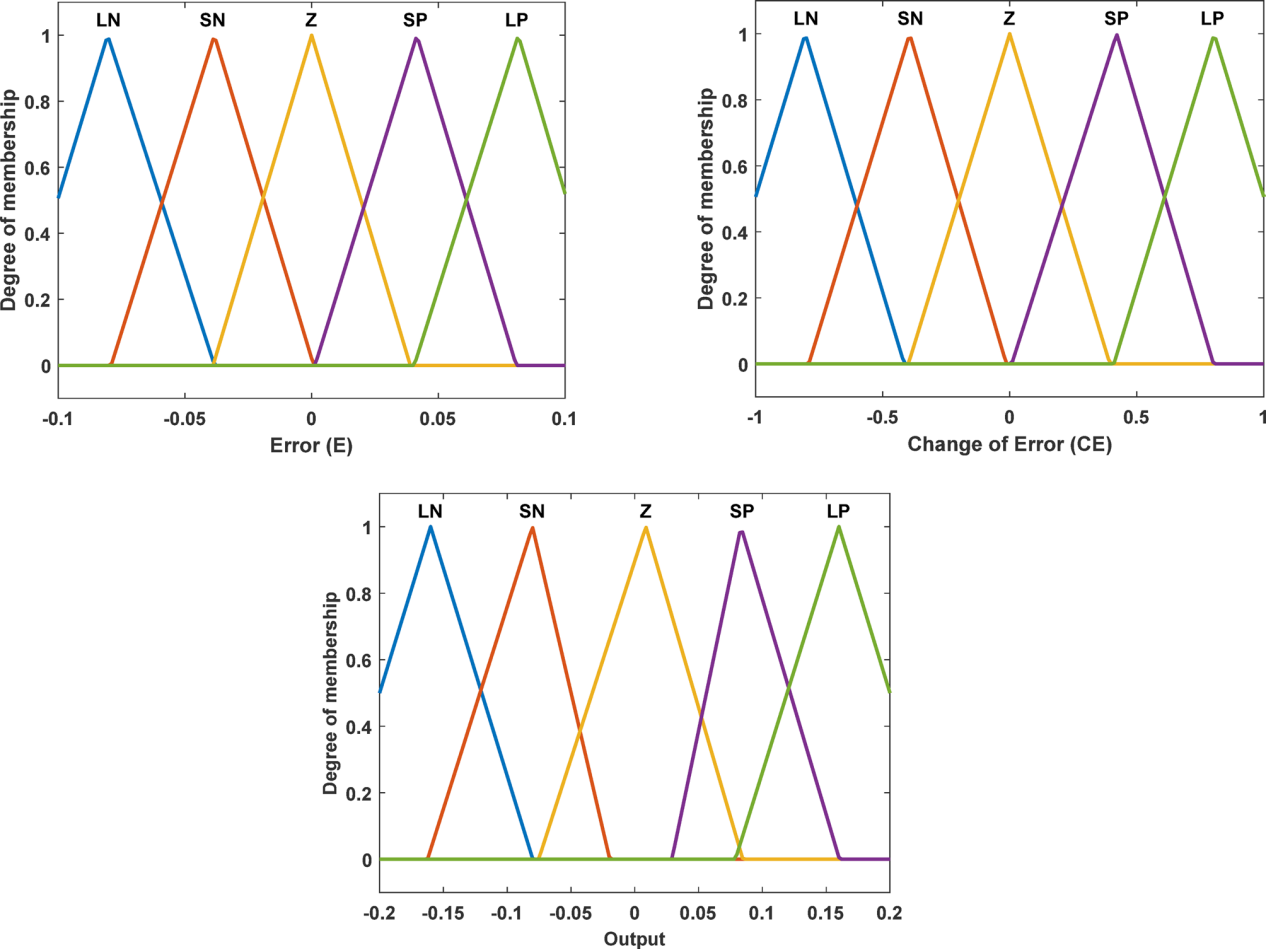
The Membership functions for fuzzy logic controllers.

The inputs and outputs of the fuzzy logic controller are both triangular membership functions, as shown in Fig. 9.

The ranges and specifications of fuzzy variable sets including (E), (CE), and (O) are presented in Table 4.

**Table 4:** Crisp range and specifications of both inputs and output variables.

Fuzzy variable	Applied MF	Interval	Crisp ranges for				
			LN	SN	Z	SP	LP
Error (E)	Triangle	[−0.1, 0.1]	[−0.12 −0.0805 −0.03835]	[−0.0791 −0.0384 0.000794]	[−0.0389 0 0.0389]	[0.00132 0.0415 0.0807]	[0.04047 0.0815 0.12]
Change of Error (CE)	Triangle	[−1, 1]	[−1.2 −0.805 −0.4153]	[−0.791 −0.394 −0.0132]	[−0.399 0 0.3942]	[0.007938 0.421 0.802]	[0.41 0.805 1.2]
Output (O)	Triangle	[−0.2, 0.2]	[−0.24 −0.16 −0.0799]	[−0.1624 −0.0802 −0.01957]	[−0.07531 0.00871 0.08469]	[0.0291 0.0832 0.1604]	[0.07885 0.16 0.24]

**Fig. 10:**
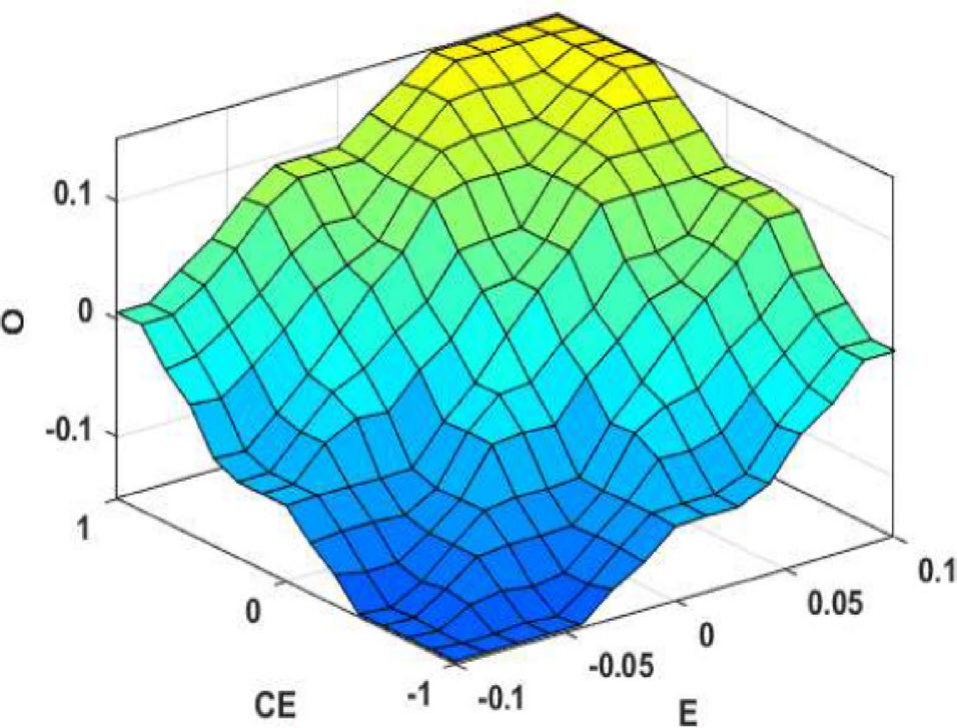
Three-dimensional rule-surface plot of the fuzzy inference system (FIS).

The fuzzy membership functions, intervals, crisp ranges, and the scaling factors (K_E_, K_CE_) were determined through a series of preliminary tuning trials conducted prior to the main simulations. These trials aimed to identify parameter values that ensured satisfactory closed-loop performance and stable system response. While the selected parameters provided adequate results for the presented studies, further systematic optimization and sensitivity analysis may improve the controller’s performance and will be addressed in future work. The rule-surface plot of the FLC Controller is shown per Fig. 10.

In the final phase, which is defuzzification, the Fuzzy Logic Controller (FLC) converts linguistic variables into crisp values. This is achieved using the Center of Gravity (COG) method, also known as the centroid technique, to generate the fuzzy output Control Law (CL).

### Optimization methods

Although several new metaheuristic algorithms have been proposed recently, the selection of PSO, GTO, and MPA in this study was motivated by their complementary search characteristics, maturity, and proven robustness in nonlinear control-optimization problems. PSO is a classical swarm-intelligence algorithm that provides rapid convergence with a well-understood parameter structure, making it an excellent benchmark for performance comparison^98^. GTO, introduced in 2021, is known for its strong balance between exploration and exploitation, which is particularly relevant to power-system control contexts^99^. MPA, on the other hand, is also a recent bio-inspired optimizer (introduced circa 2020), providing high diversity and strong convergence stability^100,101^. Using this trio allows for a fair evaluation across algorithmic generations, ensuring both benchmarking consistency and verification of tuning robustness. The focus of this work is on evaluating the proposed hybrid FPIDD²+PIDD² controller across well-established optimization frameworks rather than introducing a new algorithmic variant.

#### Particle swarm optimization (PSO)

Each particle updates its velocity and position according to:

7 $$\:{v}_{i}^{k+1}=w*{v}_{i}^{k}+{c}_{1}*{r}_{1}*\left({p}_{i}^{k}-{x}_{i}^{k}\right)+{c}_{2}*{r}_{2}*\left({g}^{k}-{x}_{i}^{k}\right)$$

8 $$\:{x}_{i}^{k+1}={x}_{i}^{k}+{v}_{i}^{k+1}$$

Where.

$\:{v}_{i}^{k}$​: Velocity of the i^th^ particle at iteration k.

$\:{x}_{i}^{k}$​: Position of the i^th^ particle.

$\:{p}_{i}^{k}$​: Best position found by the i^th^ particle.

$\:{g}^{k}$: Global best position found by the swarm.

w: The inertia weight (in this work they were set to 0.7).

c1​ & c2: The cognitive and social learning factors (in this work they were set to 2).

r1 & r2: Random numbers in [0, 1].

#### Gorilla troops optimizer (GTO)

The GTO algorithm mimics the collective and leadership behaviors of gorilla troops. The position of each agent is updated using:

9 $$\:{x}_{i}^{t+1}={X}_{i}^{t}+F*\left({X}_{leader}^{t}-{X}_{i}^{t}\right)+A*rand\left(\mathrm{0,1}\right)$$

Where.

$\:{X}_{i}^{t}$: Position of the i^th^ agent at iteration t.

$\:{X}_{leader}^{t}$​: Position of the leading agent (best solution) at iteration t.

F: Adaptive coefficient controlling the degree of movement toward the leader (in this study ∈ [0.1,0.5].

A: Parameter regulating the balance between exploration and exploitation (In this study ∈ [0,1])

rand (0,1): Uniformly distributed random number in the range [0, 1].

#### Marine predators algorithm (MPA)

The MPA simulates the foraging behavior of marine predators and employs three phases:

- Exploration (high-velocity motion).
- Transition.
- Exploitation (low-velocity motion).

The agent position update is defined as:

10 $$\:{x}_{i}^{t+1}=\left\{\begin{array}{c}{X}_{i}^{t}+P*({X}_{elite}^{t}-R*\left|{X}_{i}^{t}\right|,\:\:\:\:\:\:\:\:\:\:\:\:\:\:\:t<\frac{1}{3}{t}_{max}\\\:{X}_{i}^{t}+S*({X}_{elite}^{t}-{X}_{i}^{t},\:\:\:\:\:\:\frac{1}{3}{t}_{max}\le\:t<\frac{2}{3}{t}_{max}\\\:{X}_{i}^{t}+L*({X}_{elite}^{t}-{X}_{i}^{t}),\:\:\:\:\:\:\:\:\:\:\:\:\:\:\:\:\:\:\:\:\:\:\:t\ge\:\frac{2}{3}{t}_{max}\end{array}\right.$$

Where

$\:{X}_{i}^{t}$​: Position of the i^th^ predator at iteration t.

$\:{X}_{elite}^{t}$​: Best solution (elite predator) found so far.

P, S, and L: Random step coefficients in [0, 1] controlling the exploration and exploitation phases.

R: Random vector used to introduce stochasticity.

$\:{t}_{max}$​: Maximum number of iterations.

### Control techniques

To evaluate the performance of the proposed control strategy, simulations are conducted across four distinct case studies, each representing a different operating condition of the interconnected two-area power system. Within each case study, three experimental trials are performed, and each trial corresponds to a specific control scheme. Furthermore, within each trial, three independent runs are executed using different optimization algorithms for controller auto-tuning, including Particle Swarm Optimization (PSO), Gorilla Troops Optimizer (GTO), and Marine Predators Algorithm (MPA).

Table 5 summarizes the complete set of control techniques applied across all case studies, showing the combination of control schemes, optimization methods, and ontroller assignments for both Load Frequency Control (LFC) and Automatic Voltage Regulation (AVR) loops.

**Table 5:** Control techniques applied across all case studies.

Trail No.	Run No.	Control Scheme	Optimization Method	Controller	Controller Loop Assignment			
					LFC Loops		AVR Loops	
					$\:{LFC|}_{Area1}$	$\:{LFC|}_{Area2}$	$\:{AVR|}_{Area1}$	$\:{AVR|}_{Area2}$
1 st Trial	1 st Run	PID	PSO	PID	PID	PID	PID	PID
	2nd Run		GTO	PID	PID	PID	PID	PID
	3rd Run		MPA	PID	PID	PID	PID	PID
2nd Trial	1 st Run	PIDD²	PSO	PIDD²	PIDD²	PIDD²	PIDD²	PIDD²
	2nd Run		GTO	PIDD²	PIDD²	PIDD²	PIDD²	PIDD²
	3rd Run		MPA	PIDD²	PIDD²	PIDD²	PIDD²	PIDD²
3rd Trial	1 st Run	$\:{F{PIDD}^{2}|}_{LFC\:Loops}\:$ $\:+$ $\:\:{{PIDD}^{2}|}_{AVR\:Loops}\:$	PSO	FPIDD²+PIDD²	FPIDD²	FPIDD²	PIDD²	PIDD²
	2nd Run		GTO	FPIDD²+PIDD²	FPIDD²	FPIDD²	PIDD²	PIDD²
	3rd Run		MPA	FPIDD²+PIDD²	FPIDD²	FPIDD²	PIDD²	PIDD²

This study evaluates three distinct control schemes, as outlined in Table 5, to progressively enhance system performance. The first scheme employs conventional PID controllers in both the Load Frequency Control (LFC) and Automatic Voltage Regulation (AVR) loops, establishing a baseline for comparison. Building on this, the second scheme replaces the PID controllers with PIDD² controllers, leveraging an additional second-derivative term to improve damping and transient response. The third and proposed scheme introduces a novel hybrid configuration (FPIDD²+PIDD²), which strategically deploys FPIDD² controllers for the LFC loops and PIDD² controllers for the AVR loops.

This hybrid architecture is designed to address the distinct dynamic characteristics of each subsystem. The LFC subsystem, characterized by slower dynamics and high sensitivity to load disturbances and inter-area power exchanges, benefits from the adaptive capabilities of the FPIDD² controller. In contrast, the AVR subsystem exhibits faster dynamics, higher inherent stability margins, and limited parameter variations, where the precise and computationally efficient PIDD² controller is sufficient, and further complexity yields diminishing returns^102–105^.

In this work, the proposed control scheme is mainly founded on the PIDD² structure, where the second-derivative term provides a critical advancement over standard PID control. While the first derivative offers velocity feedback, the second derivative acts as an acceleration feedback mechanism. This enables anticipatory control, allowing the controller to preemptively counteract abrupt dynamic changes, thereby increasing the damping ratio, accelerating settling times, and enhancing robustness against disturbances and parameter variations. From a frequency-domain perspective, this introduces an additional zero, improving phase margin and mid-to-high-frequency stability^106,107^. The FPIDD² controller augments this robust foundation with fuzzy logic, enabling real-time, nonlinear adaptation of the entire control output based on instantaneous error and its derivative. This sets it apart from other advanced controllers: unlike standard Fuzzy-PID controllers that only adjust gains and lack high-frequency derivative action, FPIDD² provides full adaptive control. While Fractional-Order PID (FOPID) and ANFIS controllers can achieve high-order behavior, they are hampered by the complexity of fractional calculus and extensive training requirements, respectively^108–118^. As summarized in Table 6, the proposed FPIDD² uniquely combines high-order derivative control, real-time adaptability, and computational simplicity.

**Table 6:** Comparison of PID, Fuzzy-PID, FOPID, and FPIDD^2^ controllers.

Controller	Adaptation	Main features
PID	None	Slow transient, limited damping.
Fuzzy–PID	Gains fuzzy-tuned	No double derivative term. Limited high-frequency response.
FOPID	Fractional order	Complex tuning. May need fractional calculus.
ANFIS	Data-driven neuro-fuzzy learning	Requires extensive training datasets and high computational resources.
FPIDD² (proposed)	Fuzzy multiplies entire output	Improves transient response, damping, and adaptability without fractional calculus.

**Fig. 11:**
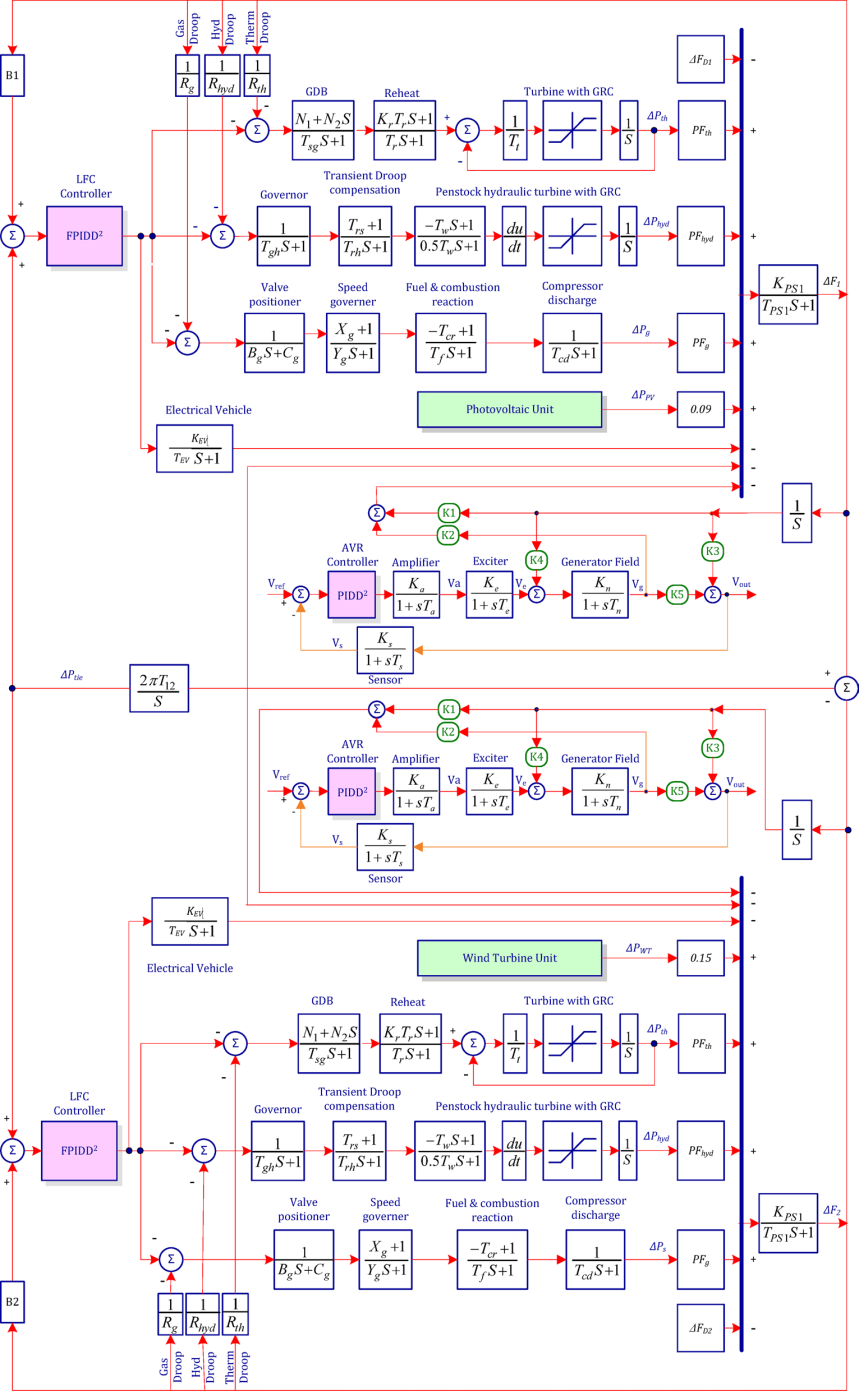
Schematic representation of the proposed hybrid FPIDD²+PIDD² control architecture for the two-area interconnected power system.

Furthermore, the hybrid FPIDD²+PIDD² framework shown in Fig. 11 delivers enhanced performance by strategically aligning controller complexity with subsystem requirements. The adaptive FPIDD² controller manages the nonlinear, time-varying dynamics of the LFC loop, while the PIDD² controller provides efficient and precise voltage regulation for the AVR loop. This selective assignment optimizes the trade-off between performance, computational cost, and implementation practicality, resulting in a robust and highly effective control strategy for interconnected power systems.

### Fitness function and boundaries

In control system design, performance indices play a crucial role in evaluating how effectively a system responds to disturbances, influencing both transient and steady-state behavior. Commonly employed indices include IAE, ISE, ITSE, and ITAE. Each index emphasizes different aspects of performance. IAE quantifies the total accumulated error, ISE penalizes large deviations more severely, ITSE highlights errors that persist over time, and ITAE assigns greater weight to errors occurring at later stages of the response. Among these, the ITAE criterion is widely regarded as the most effective due to its balanced trade-off between speed, smoothness, and stability. By penalizing prolonged errors more heavily than initial deviations, ITAE effectively minimizes sustained oscillations, reduces steady-state error, and enhances system damping. These characteristics produce a smooth and well-damped transient response with minimal overshoot and improved robustness. Moreover, ITAE-tuned systems typically yield responses that align closely with human perception of control quality, as operators prefer gradual, stable responses rather than aggressive or sluggish ones. The ITAE index also offers analytical convenience since optimal controller parameters for many standard system models have been tabulated, facilitating both theoretical and numerical optimization. Consequently, ITAE has become one of the most widely adopted objective functions in modern control engineering due to its proven ability to achieve smooth, stable, and well-damped system performance with minimal steady-state error. Accordingly, a wide range of recent control design literature deploys ITAE for LFC/AVR performance evaluation/^119,120^. In this work, the performance of each control technique is assessed using the Integral of Time-weighted Absolute Error (ITAE) index, which is mathematically expressed as:

11 $$\:ITAE={\int\:}_{0}^{{T}_{simulation}}t.(\left|\varDelta\:{F}_{1}\right|+\left|\varDelta\:{F}_{2}\right|+\left|\varDelta\:{P}_{tie}\right|+\left|\varDelta\:{V}_{1}\right|+\left|\varDelta\:{V}_{2}\right|)\:\:dt$$

The tuning parameters [K_P_, K_I_, K_D1_, N_1_, K_D2_, N_2_, K_E_, K_CE_] applied to all controllers are constrained within specified lower and upper bounds as follows:

12 $$\:\left\{\begin{array}{c}{{\mathrm{K}}_{\mathrm{P}}}_{\mathrm{m}\mathrm{i}\mathrm{n}}\le\:{\mathrm{K}}_{\mathrm{P}}\le\:{{\mathrm{K}}_{\mathrm{P}}}_{\mathrm{m}\mathrm{a}\mathrm{x}}\\\:\begin{array}{c}{{\mathrm{K}}_{\mathrm{I}}}_{\mathrm{m}\mathrm{i}\mathrm{n}}\le\:{\mathrm{K}}_{\mathrm{I}}\le\:{{\mathrm{K}}_{\mathrm{I}}}_{\mathrm{m}\mathrm{a}\mathrm{x}}\\\:{{\mathrm{K}}_{\mathrm{D}1}}_{\mathrm{m}\mathrm{i}\mathrm{n}}\le\:{\mathrm{K}}_{\mathrm{D}1}\le\:{{\mathrm{K}}_{\mathrm{D}1}}_{\mathrm{m}\mathrm{a}\mathrm{x}}\\\:\begin{array}{c}{{\mathrm{N}}_{1}}_{\mathrm{m}\mathrm{i}\mathrm{n}}\le\:{\mathrm{N}}_{1}\le\:{{\mathrm{N}}_{1}}_{\mathrm{m}\mathrm{i}\mathrm{n}}\\\:{{\mathrm{K}}_{\mathrm{D}2}}_{\mathrm{m}\mathrm{i}\mathrm{n}}\le\:{\mathrm{K}}_{\mathrm{D}2}\le\:{{\mathrm{K}}_{\mathrm{D}2}}_{\mathrm{m}\mathrm{a}\mathrm{x}}\\\:\begin{array}{c}{{\mathrm{N}}_{2}}_{\mathrm{m}\mathrm{i}\mathrm{n}}\le\:{\mathrm{N}}_{2}\le\:{{\mathrm{N}}_{2}}_{\mathrm{m}\mathrm{i}\mathrm{n}}\\\:{{\mathrm{K}}_{\mathrm{E}}}_{\mathrm{m}\mathrm{i}\mathrm{n}}\le\:{\mathrm{K}}_{\mathrm{E}}\le\:{{\mathrm{K}}_{\mathrm{E}}}_{\mathrm{m}\mathrm{a}\mathrm{x}}\\\:{{\mathrm{K}}_{\mathrm{C}\mathrm{E}}}_{\mathrm{m}\mathrm{i}\mathrm{n}}\le\:{\mathrm{K}}_{\mathrm{C}\mathrm{E}}\le\:{{\mathrm{K}}_{\mathrm{C}\mathrm{E}}}_{\mathrm{m}\mathrm{a}\mathrm{x}}\end{array}\end{array}\end{array}\end{array}\right.$$

During the optimization of controller parameters using the PSO, GTO, and MPA algorithms, the bounds were selected based on preliminary simulations to guarantee stable and practically implementable solutions. Future work may further refine these ranges to enhance convergence and robustness. The complete range of parameter boundaries used for all case studies is summarized in Table 7.

**Table 7:** Upper and lower bounds of tuning parameters for LFC and AVR systems.

Tuning Parameter	LFC Bounds		AVR Bounds		Basis for Selection
	Lower	Upper	Lower	Upper	
$\:{\mathrm{K}}_{\mathrm{P}}$	0.1	100	0.1	2	Proportional range for system stability; prevents excessive actuation
$\:{\mathrm{K}}_{\mathrm{I}}$	0.1	2	0.1	2	Ensures integral action eliminates ESS without causing slow recovery.
$\:{\mathrm{K}}_{\mathrm{D}1}$	0.1	2	0.1	2	Avoids amplification of high-frequency noise while maintaining damping.
$\:{\mathrm{N}}_{1}$	0.1	2	0.1	2	Provides adequate derivative filtering bandwidth
$\:{\mathrm{K}}_{\mathrm{D}2}$	0.1	10	0.1	2	Avoids amplification of high-frequency noise while maintaining damping.
$\:{\mathrm{N}}_{2}$	0.1	100	0.1	100	Provides adequate derivative filtering bandwidth
$\:{\mathrm{K}}_{\mathrm{E}}$	0.01	2	-	-	Maintains normalized fuzzy I/O mapping for adaptive control stability.
$\:{\mathrm{K}}_{\mathrm{C}\mathrm{E}}$	0.01	0.1	-	-	

where “–” indicates parameters not used in the AVR controller structure.

Each optimization algorithm was initialized with uniformly distributed random values within the specified bounds for all decision variables. The population size for each algorithm was set to 20 agents, and the number of iterations was fixed at 30. The stopping criterion for all optimization runs was therefore met at the end of the 30th iteration. The population size and number of iterations were determined through a series of initial trials that took place prior to the main simulations. These values were selected to achieve an appropriate balance between satisfactory optimization and reasonable computational effort. Future work will focus on developing a more systematic methodology for selecting these parameters.

## Case studies

### Case study no. 1: effect of step load perturbations

#### System description

This case study is exactly the same as the two-area interconnected power system illustrated in Fig. 11, but without any renewable energy sources. In addition, 2.5% SLP injected in the second area at 150 s and 2.5% SLP injected into the first area at 50 s.

#### Simulation results

**Fig. 12:**
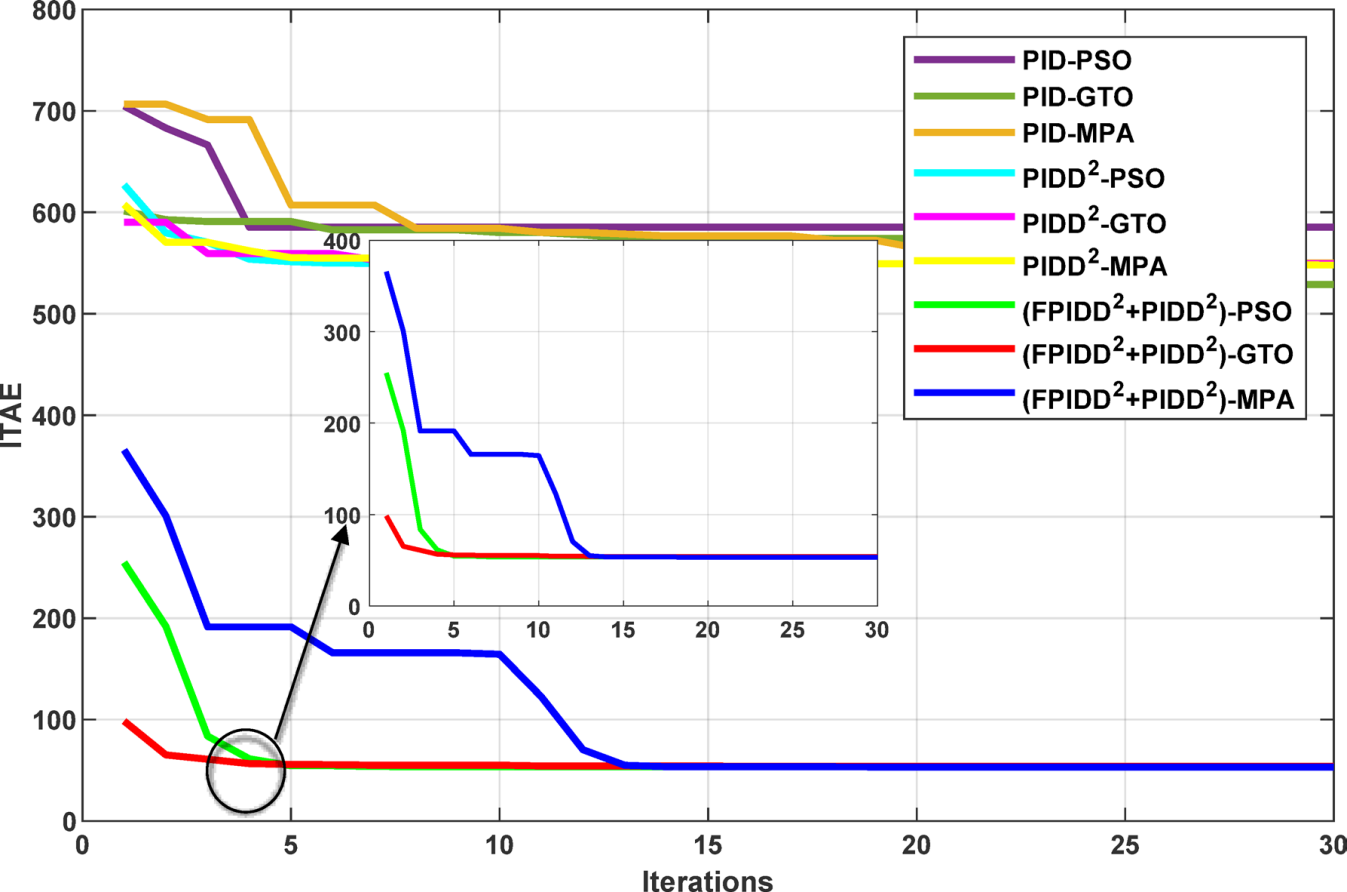
Convergence behavior of the ITAE during controller optimization in Case Study No.1. The curves correspond to: PID–PSO (Purple), PID–GTO (Olive Green), PID–MPA (Light Brown), PIDD²–PSO (Cyan), PIDD²–GTO (Magenta), PIDD²–MPA (Yellow), (FPIDD²+PIDD²)–PSO (Light Green), (FPIDD²+PIDD²)–GTO (Red), and (FPIDD²+PIDD²)–MPA (Blue).

**Fig. 13:**
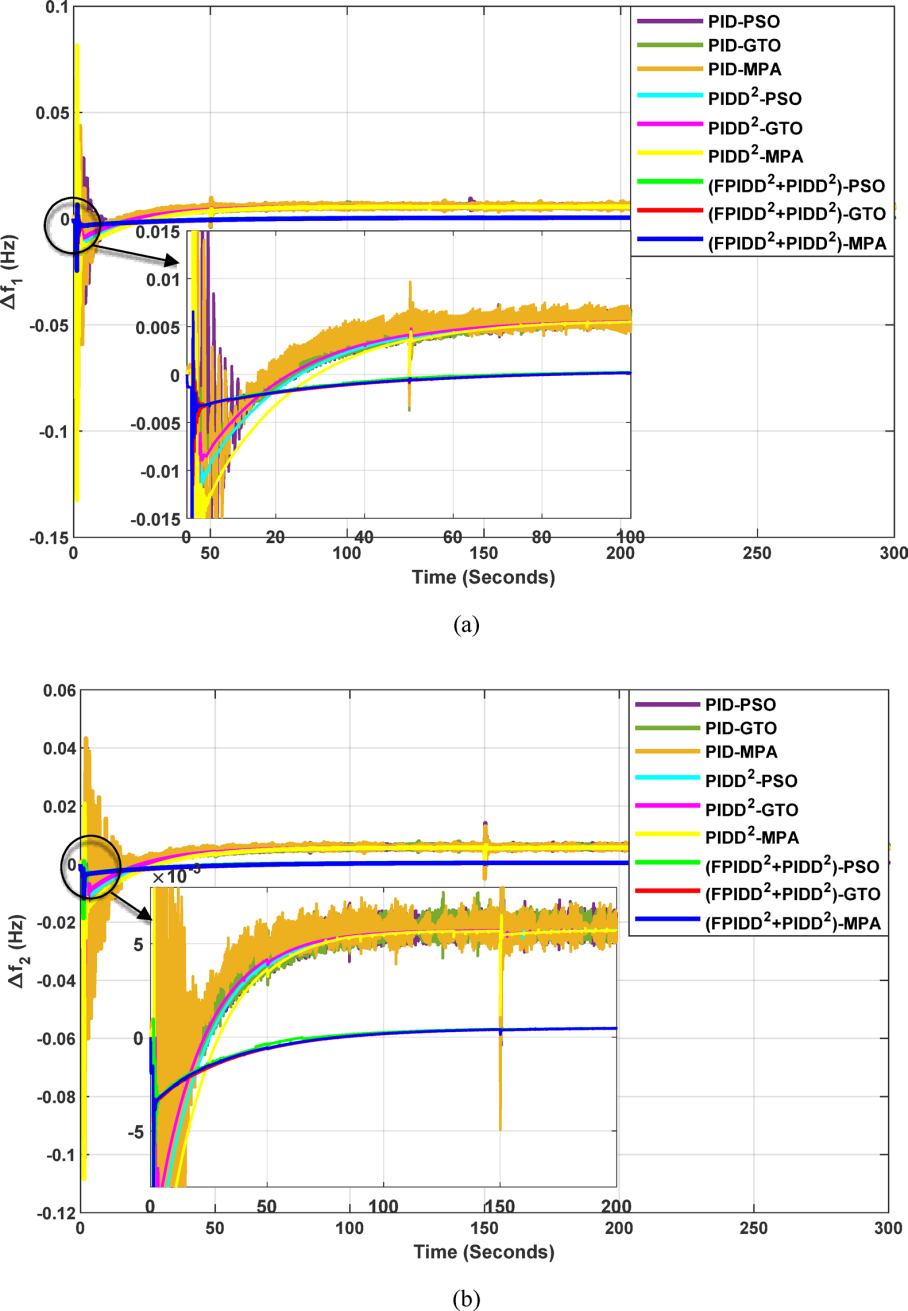

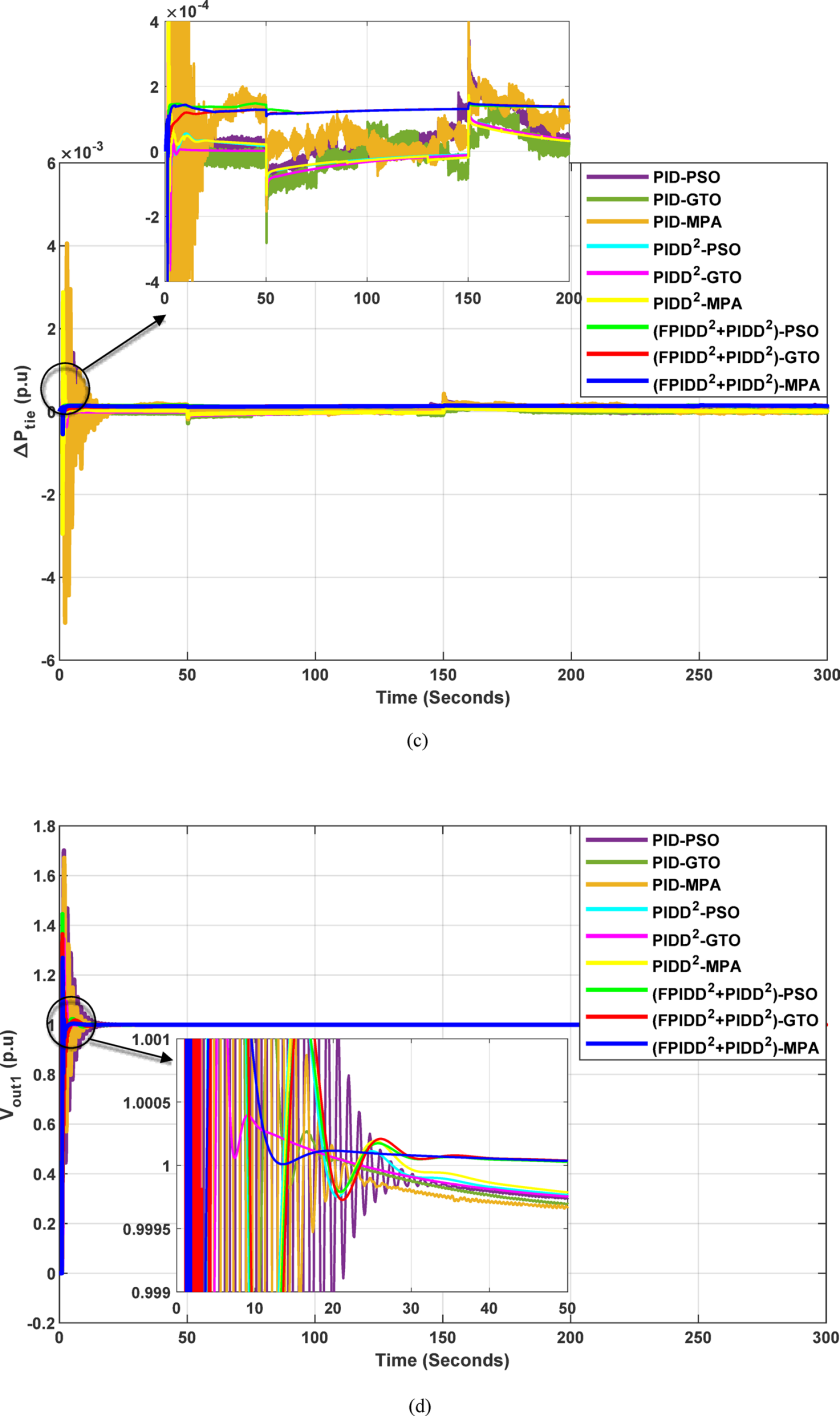

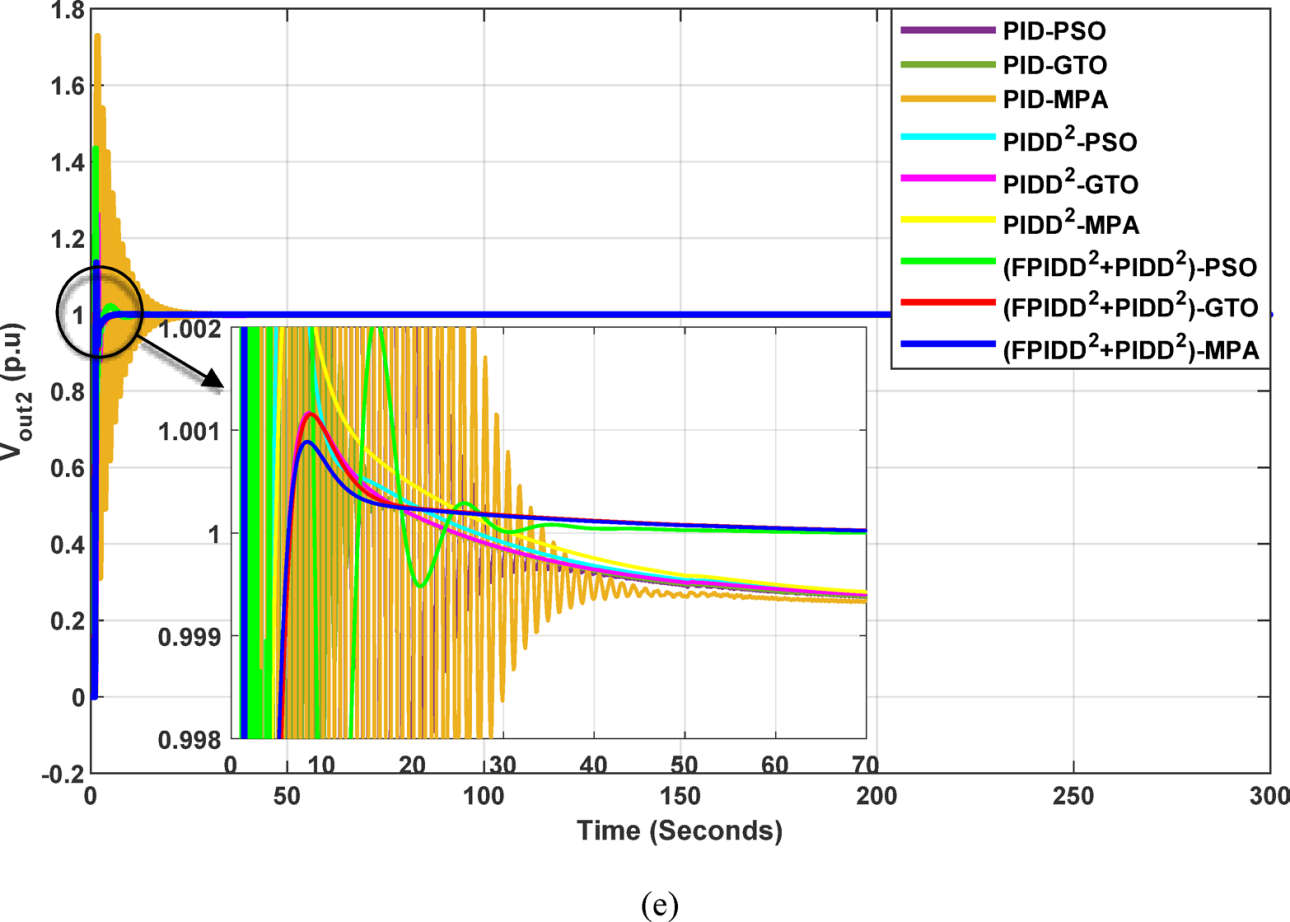
Dynamic responses of the two-area system of Case Study No.1. **a** ∆f₁, **b** ∆f₂, **c** ΔP_tie_, **d** V_out1_, **e** V_out2_. The curves correspond to: PID–PSO (Purple), PID–GTO (Olive Green), PID–MPA (Light Brown), PIDD²–PSO (Cyan), PIDD²–GTO (Magenta), PIDD²–MPA (Yellow), (FPIDD²+PIDD²)–PSO (Light Green), (FPIDD²+PIDD²)–GTO (Red), and (FPIDD²+PIDD²)–MPA (Blue).

The convergence curves of the Integral Time Absolute Error (ITAE) are shown along with Time Absolute Error trends are illustrated in Fig. 12, while the power system dynamic responses are presented in Fig. 13.

Figure 12 shows that almost all control schemes start with relatively high ITAE values, indicating substantial system error prior to optimization. Among them, the proposed FPIDD²+PIDD² control scheme, particularly when tuned using the MPA algorithm, exhibits the most rapid and significant improvement, reducing the ITAE from approximately 350 to below 50 within the first 15 iterations. When optimized using PSO and GTO, the same controller also demonstrates strong performance, achieving almost the same ITAE. In contrast, both the conventional PID and PIDD² controllers converge more slowly and settle at considerably higher ITAE values, ranging from approximately 530 to 590, indicating lower effectiveness in minimizing dynamic error. These results clearly confirm that the FPIDD²+PIDD² controller offers superior dynamic performance and enhanced system stability, consistently achieving the lowest ITAE across all tested configurations.

As illustrated in Fig. 10, the dynamic responses of frequency deviation in Area-1 (∆f_1_) and Area-2 (∆f_2_) reveal distinct performance differences among the examined control schemes. The conventional PID controllers (dark purple, dark green, and brown curves) exhibit pronounced oscillations and large undershoots following the load disturbance, indicating weaker damping characteristics and slower recovery. The PIDD² controllers (cyan, magenta, and yellow curves) improve system stability by reducing oscillation amplitude, resulting in a faster and more stable transition toward steady state.

In contrast, the proposed FPIDD²+PIDD² configuration (green, red, and blue curves) delivers markedly superior performance. It demonstrates the most rapid and well-damped frequency response, with negligible overshoot and the shortest settling time. This reflects enhanced damping capability, faster disturbance rejection, and robust inter-area coordination, ensuring superior frequency stabilization across the interconnected system. Regarding the tie-line power deviation (∆Ptie), the conventional PID curves show the largest oscillation amplitudes, indicating limited synchronization capability and higher power exchange fluctuations during transients. The PIDD² controllers significantly mitigate these oscillations and achieve faster convergence; however, small steady-state deviations remain observable. Conversely, the proposed FPIDD²+PIDD² controller provides the most stable and well-damped tie-line response, achieving smooth and coordinated power exchange between interconnected areas while maintaining overall transient stability. For the voltage responses in Area-1 (V_out1_) and Area-2 (V_out2_), all controllers initially exhibit transient behavior before settling near the nominal voltage of 1.0 p.u. Nonetheless, the response characteristics differ considerably among the control schemes. The proposed FPIDD²+PIDD² control approach achieves the fastest settling time, minimal overshoot, and smoothest transient profile. In contrast, the conventional PID and PIDD² controllers, regardless of the optimization algorithm employed, display higher overshoot and slower voltage recovery, showing the most pronounced overshoot and sluggish damping. Overall, these findings confirm that the FPIDD²+PIDD² control structure significantly enhances both frequency and voltage regulation performance by providing faster settling, reduced overshoot, improved damping, and superior robustness under load disturbances.

The optimal controllers’ sittings obtained for case study No.1 are shown per Table 8, and Table 9, while the power system dynamic responses are presented in Table 10.

**Table 8:** Optimal controllers’ settings for the LFC loop in the first and second area.

Controller	Optim. Algorithm	$\:{LFC|}_{Area1}$								$\:{LFC|}_{Area2}$							
		K_*P*_	K_I_	K_D1_	*N*_1_	K_D2_	*N*_2_	K_E_	K_CE_	K_*P*_	K_I_	K_D1_	*N*_1_	K_D2_	*N*_2_	K_E_	K_CE_
PID	PSO	97.9	2.0	2.0	1.7	-	-	-	-	100.0	2.0	1.9	2.0	-	-	-	-
PID	GTO	99.9	2.0	2.0	2.0	-	-	-	-	99.9	2.0	0.7	2.0	-	-	-	-
PID	MPA	63.4	2.0	1.2	2.0	-	-	-	-	76.1	2.0	1.5	1.8	-	-	-	-
PIDD²	PSO	100.0	2.0	2.0	2.0	10.0	100.0	-	-	100.0	2.0	1.7	2.0	10.0	100.0	-	-
PIDD²	GTO	100.0	2.0	1.4	2.0	10.0	33.9	-	-	100.0	2.0	2.0	1.7	7.5	50.7	-	-
PIDD²	MPA	99.5	2.0	0.7	2.0	10.0	100.0	-	-	100.0	2.0	0.8	0.7	9.9	98.8	-	-
FPIDD²+PIDD²	PSO	100.0	2.0	2.0	2.0	8.5	100.0	2.0	0.1	100.0	2.0	2.0	2.0	10.0	100.0	2.0	0.1
FPIDD²+PIDD²	GTO	100.0	2.0	1.7	1.9	10.0	91.1	2.0	0.1	100.0	2.0	2.0	0.7	10.0	100.0	2.0	0.0
FPIDD²+PIDD²	MPA	100.0	2.0	1.9	2.0	2.3	100.0	2.0	0.1	100.0	2.0	2.0	2.0	10.0	92.8	2.0	0.0

**Table 9:** Optimal controllers’ settings for the AVR loop in the first and second area.

Controller	Optim. Algorithm	$\:{AVR|}_{Area1}$								$\:AVR{|}_{Area2}$							
		K_*P*_	K_I_	K_D1_	*N*_1_	K_D2_	*N*_2_	K_E_	K_CE_	K_*P*_	K_I_	K_D1_	*N*_1_	K_D2_	*N*_2_	K_E_	K_CE_
PID	PSO	0.7	2.0	1.4	1.1	-	-	-	-	1.7	1.4	2.0	0.9	-	-	-	-
PID	GTO	0.4	1.7	2.0	0.3	-	-	-	-	2.0	1.7	1.9	0.2	-	-	-	-
PID	MPA	0.1	1.9	1.3	1.7	-	-	-	-	0.2	2.0	2.0	1.6	-	-	-	-
PIDD²	PSO	2.0	2.0	1.9	2.0	2.0	6.7	-	-	2.0	2.0	2.0	2.0	2.0	100.0	-	-
PIDD²	GTO	1.4	2.0	0.3	0.7	1.5	8.8	-	-	1.7	1.8	1.0	1.2	0.5	95.9	-	-
PIDD²	MPA	2.0	2.0	2.0	2.0	2.0	99.8	-	-	2.0	1.1	0.1	2.0	1.6	94.2	-	-
FPIDD²+PIDD²	PSO	2.0	2.0	2.0	2.0	1.9	100.0	-	-	1.8	2.0	2.0	2.0	2.0	100.0	-	-
FPIDD²+PIDD²	GTO	2.0	2.0	2.0	2.0	2.0	100.0	-	-	2.0	2.0	1.4	2.0	1.1	100.0	-	-
FPIDD²+PIDD²	MPA	2.0	2.0	2.0	2.0	0.7	98.4	-	-	0.5	0.2	1.9	1.6	1.0	95.4	-	-

**Table 10:** Dynamic responses of the system.

Controller	Optim. Algorithm	∆f_1_		∆f_2_		∆*P*_tie_		V_out1_			V_out2_		
		MO_∆f1_ (Hz)	MU_∆f1_ (Hz)	MO_∆f2_ (Hz)	MU_∆f2_ (Hz)	MO_∆*P*_ (*p*.u)	MU_∆*P*_ (*p*.u)	MP_− V1_ (*p*.u)	T_*r*−V1_ (Sec.)	T_s−V1_ (Sec.)	MP_− V2_ (*p*.u)	T_*r*−V2_ (Sec.)	T_s−V2_ (Sec.)
PID	PSO	0.033	−0.068	0.031	−0.069	0.001	−0.002	0.701	0.269	11.923	0.694	0.252	14.215
PID	GTO	0.009	−0.055	0.021	−0.064	0.001	−0.001	0.302	0.312	5.699	0.585	0.267	7.008
PID	MPA	0.044	−0.090	0.043	−0.103	0.004	−0.005	0.672	0.319	9.362	0.728	0.250	17.350
PIDD²	PSO	0.010	−0.066	0.006	−0.063	0.001	−0.001	0.412	0.070	3.452	0.164	0.144	3.204
PIDD²	GTO	0.006	−0.049	0.006	−0.046	0.000	0.000	0.345	0.238	2.884	0.262	0.246	3.338
PIDD²	MPA	0.081	−0.132	0.021	−0.108	0.003	−0.003	0.376	0.068	3.700	0.177	0.157	3.012
FPIDD²+PIDD²	PSO	0.004	−0.021	0.001	−0.019	0.000	0.000	0.388	0.069	3.451	0.359	0.067	3.439
FPIDD²+PIDD²	GTO	0.004	−0.021	0.001	−0.012	0.000	0.000	0.364	0.067	3.645	0.136	0.197	2.948
FPIDD²+PIDD²	MPA	0.007	−0.024	0.001	−0.012	0.000	−0.001	0.269	0.135	2.143	0.136	0.206	2.792

Table 10 demonstrates that the proposed FPIDD²+PIDD² controller consistently outperforms both conventional PID and PIDD² controllers across all tested optimization algorithms.

Regarding frequency deviation, the FPIDD²+PIDD² scheme achieves the lowest overshoot and undershoot in both control areas. For example, under MPA optimization, the peak frequency deviations for Area-1 and Area-2 (∆f₁ and ∆f₂) are limited to 0.0066 Hz and 0.0005 Hz, respectively. Similarly, the tie-line power deviation peaks at only 0.0002 p.u., indicating excellent inter-area stability.

In terms of voltage response, the FPIDD²+PIDD² controller also exhibits superior performance, delivering the fastest settling times and the lowest overshoot. Specifically, with MPA optimization, V_out1_ settles in 2.14 s and _Vout2_ in 2.79 s. Moreover, the voltage transients are smoother, with minimal overshoot value 0.2692 p.u. for V_out1_ and 0.1358 p.u. for V_out2_, highlighting the controller’s effectiveness in managing dynamic voltage behavior.

The Integral Time Absolute Error (ITAE) values for each of the three control schemes, tuned using the three optimization algorithms, are presented in Table 11.

**Table 11:** The optimism integral time absolute error (ITAE) for all controllers.

Controller	Optimization Algorithm			Best ITAE	
	PSO	GTO	MPA	Value	Algorithm
PID	585.24	528.84	550.15	528.84	GTO
PIDD²	549.29	549.37	548.02	548.02	MPA
FPIDD²+ PIDD²	53.64	53.91	53.21	53.21	MPA

Table 11 shows that the proposed FPIDD²+PIDD² control scheme achieves a significant improvement over both the PID and PIDD² controllers under all optimization algorithms. Notably, when tuned using the MPA algorithm, the ITAE was reduced sharply reaching nearly one-tenth of the values achieved by the PID and PIDD² controllers.

#### Results summary

Although traditional PID controllers delivered acceptable dynamic performance in this case study, they were limited by considerable overshoot and prolonged settling times, resulting in a best-achieved Integral of Time-weighted Absolute Error (ITAE) of approximately 529. In comparison, the PIDD² controllers provided a marginal improvement, with the MPA yielding a modest 3.6% reduction in ITAE.

In contrast, the proposed hybrid control configuration (FPIDD²+PIDD²) demonstrated superior performance across all evaluated metrics. This setup achieved minimal steady-state error, reduced overshoot, faster rise times, and shorter settling durations. Notably, the hybrid scheme tuned by MPA led to a significant reduction in ITAE: 89.9% compared to the PID controllers and 90.3% compared to the PIDD² controllers, confirming its enhanced control accuracy and system robustness.

Furthermore, the analysis of optimization techniques revealed that for PID controllers, MPA offered a slight advantage over the Gorilla Troops Optimizer (GTO), which in turn marginally outperformed Particle Swarm Optimization (PSO). However, for both the PIDD² and FPIDD²+PIDD² controllers, all three optimization methods produced nearly identical results. This suggests that when advanced control structures are employed, the controller architecture has a more pronounced impact on system performance than the specific optimization algorithm used.

Collectively, these findings demonstrate that the FPIDD²+PIDD² hybrid control strategy is the most effective solution for load frequency and voltage regulation in multi-area power systems, offering a level of precision, stability, and responsiveness that surpasses both conventional and intermediate designs.

### Case study no. 2: effect of renewable energy sources (PV + WT)

#### System description

This case study is exactly the same as the two-area interconnected power system illustrated in Fig. 4, but with a Photovoltaic unit is connected to the first area after 20 s and a wind turbine a unit is inserted to the second area at 70 s. In addition, 2.5% SLP injected in the second area at 150 s and 2.5% SLP injected to the first area at 50 s.

#### Simulation results

**Fig. 14:**
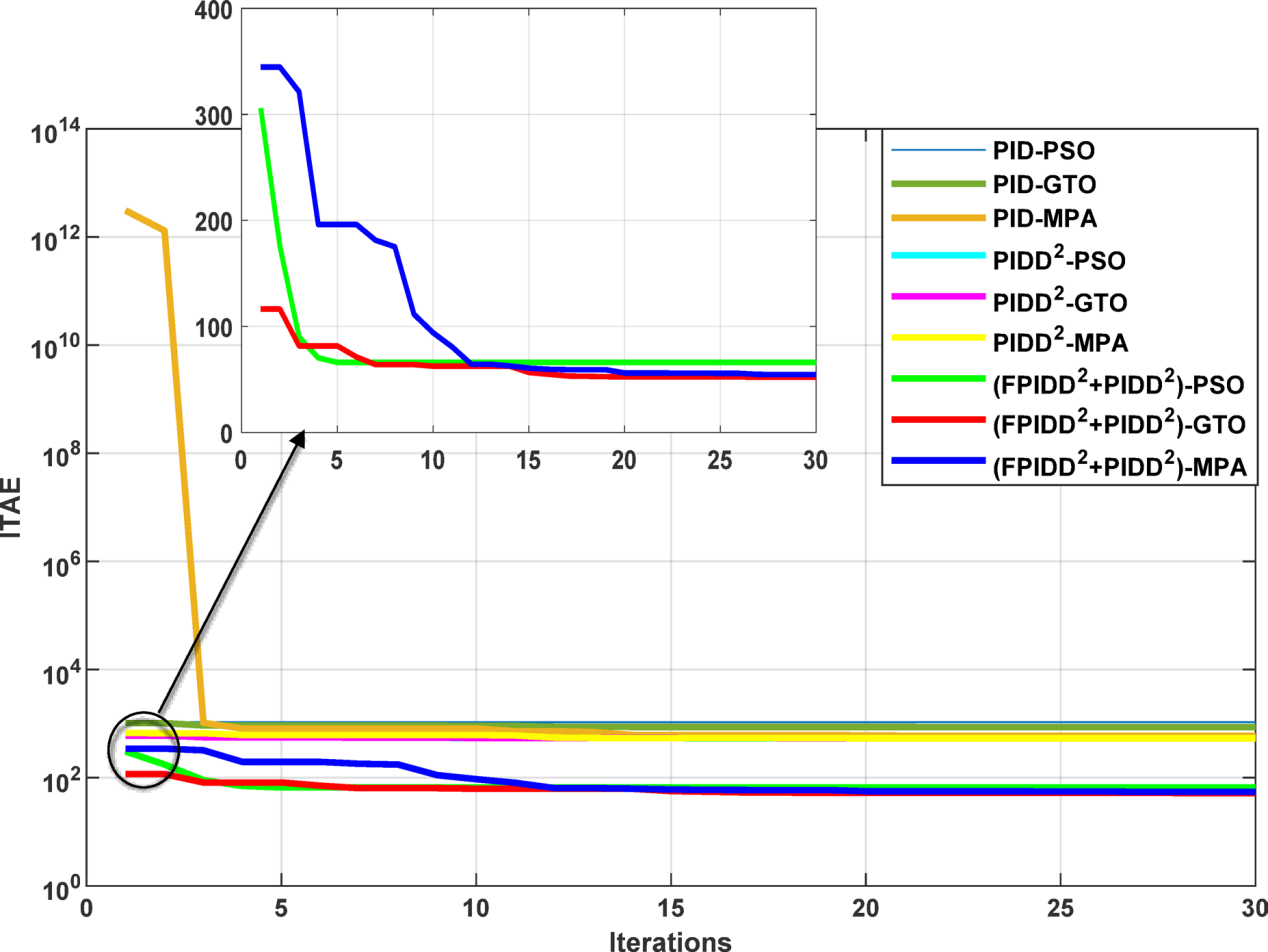
Convergence behavior of the ITAE during controller optimization in Case Study No. 2. The curves correspond to: PID–PSO (Purple), PID–GTO (Olive Green), PID–MPA (Light Brown), PIDD²–PSO (Cyan), PIDD²–GTO (Magenta), PIDD²–MPA (Yellow), (FPIDD²+PIDD²)–PSO (Light Green), (FPIDD²+PIDD²)–GTO (Red), and (FPIDD²+PIDD²)–MPA (Blue).

**Fig. 15:**
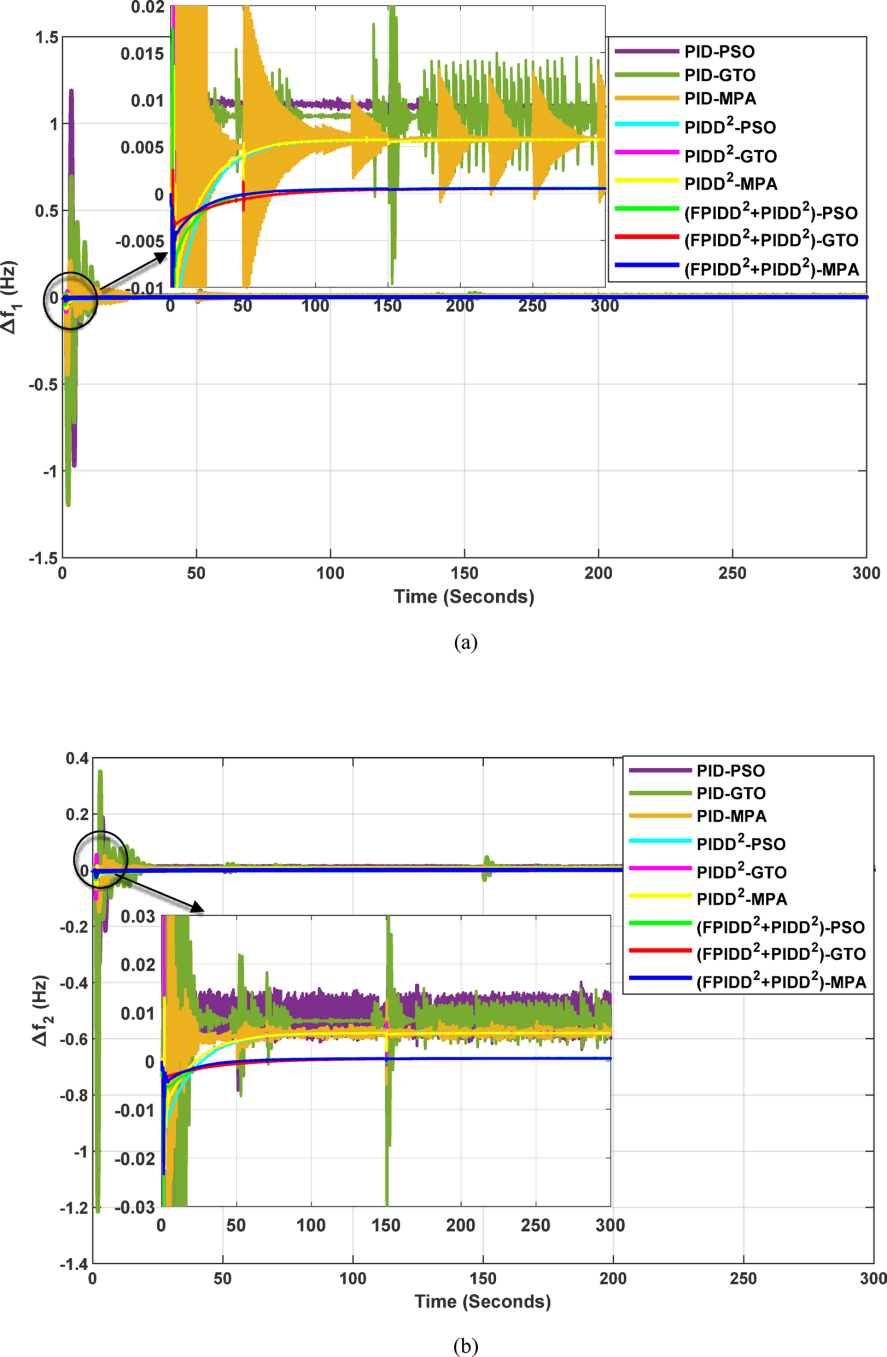

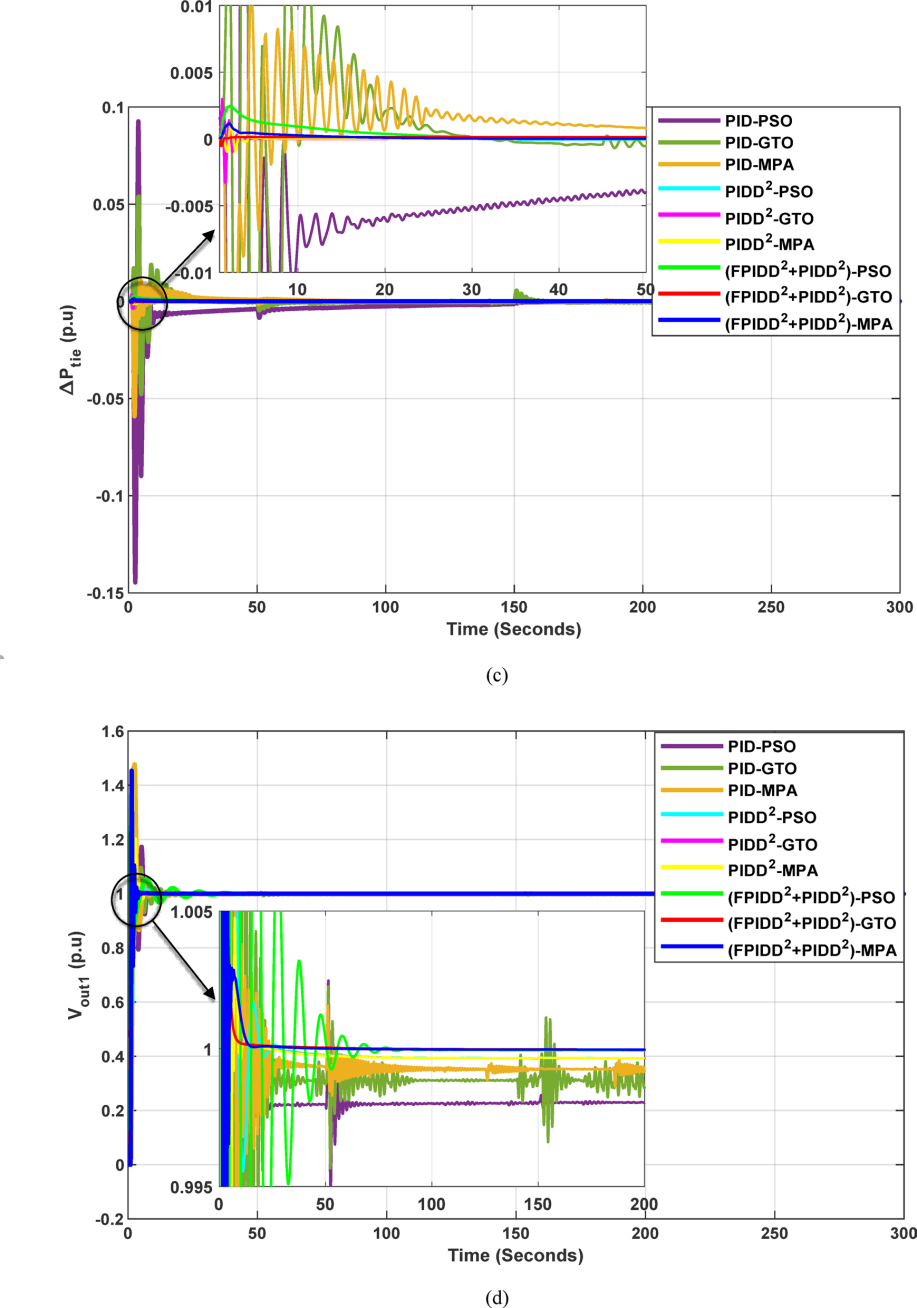

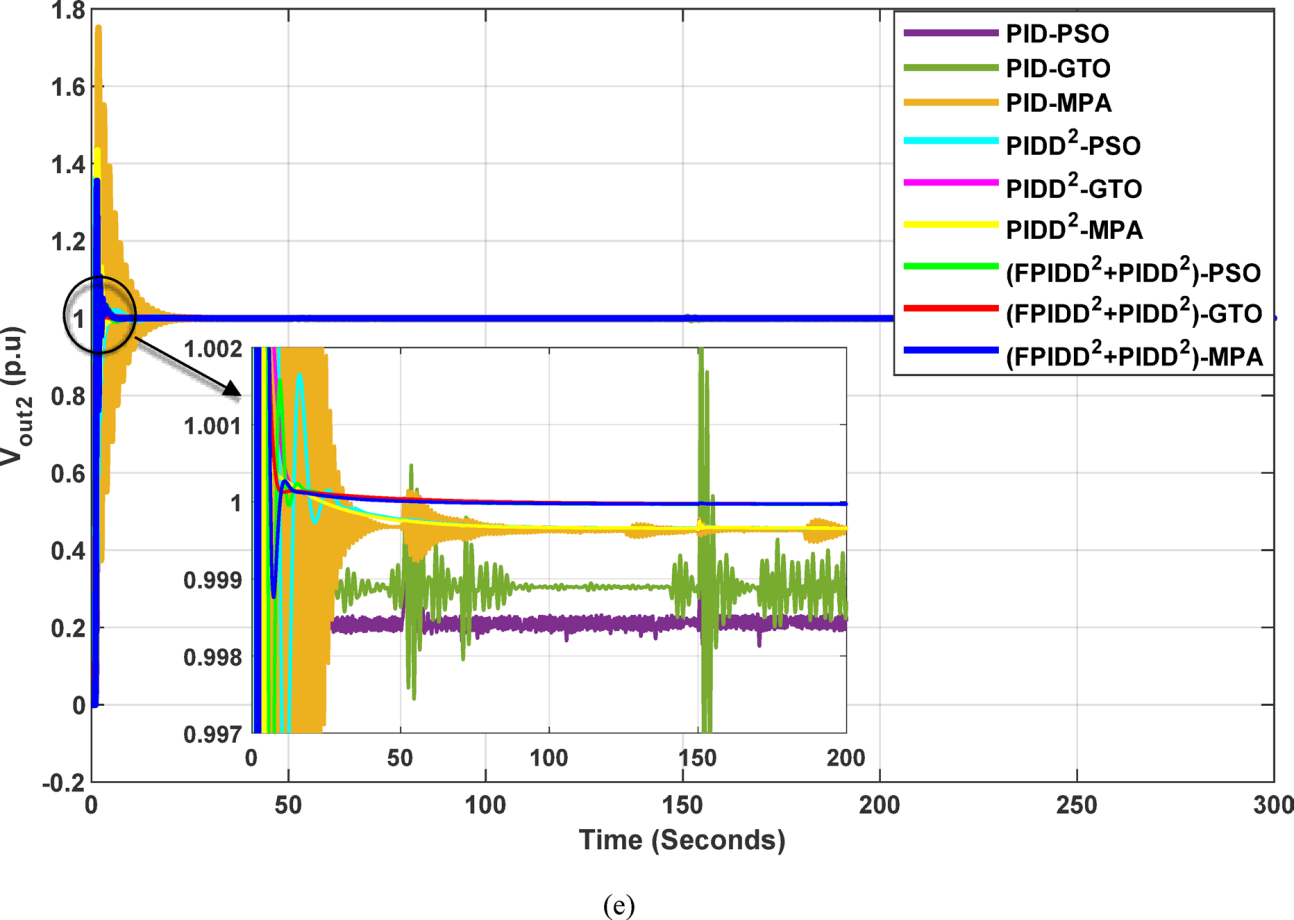
Dynamic responses of the two-area system of Case Study No.2: **a** ∆f₁, **b** ∆f₂, **c** ΔP_tie_, **d** V_out1_, **e** V_out2_. The curves correspond to: PID–PSO (Purple), PID–GTO (Olive Green), PID–MPA (Light Brown), PIDD²–PSO (Cyan), PIDD²–GTO (Magenta), PIDD²–MPA (Yellow), (FPIDD²+PIDD²)–PSO (Light Green), (FPIDD²+PIDD²)–GTO (Red), and (FPIDD²+PIDD²)–MPA (Blue).

The convergence curves of the Integral Time Absolute Error (ITAE) are shown along with Time Absolute Error trends are illustrated in Fig. 14, while the power system dynamic responses are presented in Fig. 15.

Figure 14 demonstrates that the hybrid (FPIDD²+PIDD²) controllers consistently outperform both conventional PID and second-order PIDD² structures in terms of convergence speed and final ITAE value. These controllers achieve the lowest ITAE levels while exhibiting smooth and stable convergence behavior. While PIDD²-based designs offer moderate improvements over classical PID, they still fall short of the performance achieved by the hybrid structure. In contrast, traditional PID configurations display relatively poor convergence, likely due to instability in initial parameter tuning or sensitivity to the optimization algorithm. Overall, the figure highlights that the (FPIDD²+PIDD²) controllers, particularly when optimized using advanced metaheuristic algorithms, significantly enhance tuning accuracy and dynamic response, resulting in superior performance in minimizing ITAE and accelerating convergence during control parameter optimization.

As illustrated in Fig. 15, the dynamic response of the system frequency deviation reveals clear distinctions among the three control strategies. The conventional PID controllers exhibit the largest oscillations and the and the highest peaks, indicating limited damping capability and reduced robustness against inter-area load disturbances in both areas. This behavior reflects their weak resilience to dynamic fluctuations and limited ability to suppress frequency deviations effectively. The PIDD² controllers yields a noticeable improvement, characterized by reduced overshoot and smaller oscillation amplitudes. Nevertheless, these controllers still require a relatively long duration to reach steady-state conditions, suggesting that damping performance, while enhanced, remains suboptimal. In contrast, the proposed FPIDD²+PIDD² control scheme demonstrates the most stable frequency response with minimal overshoot. This superior transient performance highlights its strong damping capability and enhanced robustness under varying load conditions.

Regarding the tie-line power deviation (∆Ptie), the conventional PID controllers produce the largest oscillations and the highest peaks, indicating poor regulation of inter-area power exchange and limited damping effectiveness. The PIDD² controllers improve performance by reducing the magnitude of oscillations as well as overshoots, though transient ripples remain noticeable. In comparison, the proposed FPIDD²+PIDD² controllers achieve the most efficient tie-line response, characterized by minimal overshoot, smooth transients, and rapid convergence to steady state. These attributes lead to improved power flow regulation and stronger synchronization between interconnected areas. The enhanced damping and negligible steady-state error confirm the effectiveness of the proposed approach in maintaining tie-line power stability under load disturbances.

With respect to voltage performance, all controllers initially exhibit a sharp rise in response to the applied step disturbance. The conventional PID-based schemes show pronounced overshoots and oscillations before eventually settling at the nominal value. The PIDD²-based controllers demonstrate improved damping and reduced overshoot compared with the conventional PID group, though minor oscillations persist before steady state is reached, indicating only moderate enhancement in robustness. Conversely, the proposed hybrid FPIDD²+PIDD² configuration exhibits the most stable and well-damped voltage response. The output voltage swiftly returns to unity with negligible overshoot and minimal oscillations. Among all configurations, reflecting superior dynamic performance and effective voltage regulation under disturbance conditions.

In this case study, the optimal controllers’ gains obtained for case study No.2 are shown per Table 12, and Table 13, while the power system dynamic responses are presented in Table 14.

**Table 12:** Optimal controllers’ settings for the LFC loop in the first and second area.

Controller	Optim. Algorithm	$\:{LFC|}_{Area1}$								$\:{LFC|}_{Area2}$							
		K_*P*_	K_I_	K_D1_	*N*_1_	K_D2_	*N*_2_	K_E_	K_CE_	K_*P*_	K_I_	K_D1_	*N*_1_	K_D2_	*N*_2_	K_E_	K_CE_
PID	PSO	0.1	0.1	0.1	0.3	-	-	-	-	17.4	0.2	0.2	1.4	-	-	-	-
PID	GTO	0.1	0.6	0.6	0.5	-	-	-	-	0.1	0.2	0.5	0.2	-	-	-	-
PID	MPA	3.2	2.0	1.7	0.9	-	-	-	-	86.2	1.9	1.9	0.3	-	-	-	-
PIDD²	PSO	100.0	2.0	2.0	2.0	10.0	100.0	-	-	100.0	2.0	2.0	2.0	10.0	100.0	-	-
PIDD²	GTO	99.6	2.0	2.0	0.1	10.0	100.0	-	-	100.0	2.0	2.0	0.2	1.9	100.0	-	-
PIDD²	MPA	100.0	2.0	0.3	2.0	10.0	100.0	-	-	99.8	2.0	1.0	1.9	10.0	99.8	-	-
FPIDD²+PIDD²	PSO	100.0	2.0	1.0	2.0	6.4	100.0	2.0	0.1	44.0	2.0	2.0	0.7	9.6	75.4	1.1	0.1
FPIDD²+PIDD²	GTO	100.0	2.0	0.7	2.0	7.1	0.1	2.0	0.1	100.0	2.0	2.0	2.0	10.0	100.0	2.0	0.0
FPIDD²+PIDD²	MPA	80.1	2.0	2.0	2.0	9.9	100.0	2.0	0.1	44.8	2.0	2.0	0.4	10.0	99.1	2.0	0.1

**Table 13:** Optimal controllers’ settings for the AVR loop in the first and second area.

Controller	Optim. algorithm	$\:{AVR|}_{Area1}$								$\:AVR{|}_{Area2}$							
		K_*P*_	K_I_	K_D1_	*N*_1_	K_D2_	*N*_2_	K_E_	K_CE_	K_*P*_	K_I_	K_D1_	*N*_1_	K_D2_	*N*_2_	K_E_	K_CE_
PID	PSO	0.5	0.6	0.1	1.9	-	-	-	-	0.7	0.7	0.2	1.4	-	-	-	-
PID	GTO	0.7	0.9	0.2	0.5	-	-	-	-	0.6	0.9	0.7	0.7	-	-	-	-
PID	MPA	0.2	0.9	0.4	0.7	-	-	-	-	0.9	1.9	1.2	1.8	-	-	-	-
PIDD²	PSO	2.0	2.0	2.0	2.0	2.0	100.0	-	-	2.0	2.0	2.0	2.0	2.0	100.0	-	-
PIDD²	GTO	1.7	2.0	1.5	0.2	0.4	7.6	-	-	2.0	2.0	1.4	2.0	0.6	100.0	-	-
PIDD²	MPA	0.8	2.0	2.0	0.3	2.0	0.1	-	-	2.0	2.0	2.0	0.1	2.0	0.9	-	-
FPIDD²+PIDD²	PSO	1.0	1.3	2.0	1.3	2.0	0.0	-	-	1.5	2.0	1.0	1.2	2.0	100.0	-	-
FPIDD²+PIDD²	GTO	2.0	2.0	0.8	2.0	1.1	0.0	-	-	1.9	2.0	1.1	1.4	0.7	100.0	-	-
FPIDD²+PIDD²	MPA	2.0	2.0	2.0	2.0	0.2	77.4	-	-	0.8	2.0	1.4	2.0	0.6	14.9	-	-

**Table 14:** Dynamic responses of the system.

Controller	Optim. Algorithm	∆f_1_		∆f_2_		∆*P*_tie_		V_out1_			V_out2_		
		MO_∆f1_ (Hz)	MU_∆f1_ (Hz)	MO_∆f2_ (Hz)	MU_∆f2_ (Hz)	MO_∆*P*_ (*p*.u)	MU_∆*P*_ (*p*.u)	MP_− V1_ (*p*.u)	T_*r*−V1_ (Sec.)	T_s−V1_ (Sec.)	MP_− V2_ (*p*.u)	T_*r*−V2_ (Sec.)	T_s−V2_ (Sec.)
PID	PSO	1.188	−0.977	0.188	−0.332	0.093	−0.145	0.379	0.543	9.133	0.398	0.430	6.311
PID	GTO	0.695	−1.196	0.350	−1.214	0.054	−0.048	0.465	0.432	11.602	0.364	0.386	5.230
PID	MPA	0.208	−0.445	0.049	−0.146	0.011	−0.059	0.477	0.558	6.601	0.751	0.268	16.110
PIDD²	PSO	0.010	−0.065	0.010	−0.065	0.000	0.000	0.364	0.067	3.703	0.364	0.067	3.703
PIDD²	GTO	0.033	−0.083	0.053	−0.102	0.003	−0.003	0.314	0.200	2.593	0.248	0.171	1.891
PIDD²	MPA	0.014	−0.063	0.013	−0.065	0.001	−0.001	0.444	0.257	4.721	0.434	0.188	3.356
FPIDD²+PIDD²	PSO	0.018	−0.041	0.001	−0.031	0.000	0.000	0.315	0.068	17.796	0.153	0.154	4.311
FPIDD²+PIDD²	GTO	0.003	−0.022	0.001	−0.012	0.000	−0.001	0.179	0.174	1.824	0.191	0.186	1.873
FPIDD²+PIDD²	MPA	0.001	−0.014	0.001	−0.023	0.001	0.000	0.454	0.191	3.263	0.355	0.181	4.112

The data presented in Table 14 clearly indicate that the hybrid FPIDD²+PIDD² controller offers superior dynamic performance compared to both conventional PID and second-order PIDD² controllers. This advanced structure enhances transient stability, effectively suppresses oscillations, and enables faster recovery following disturbances, resulting in a well-damped and stable system response. In terms of frequency deviation, the FPIDD²+PIDD² controller consistently achieves the lowest overshoot and undershoot values. Notably, when optimized using the GTO algorithm, it limits ∆f_1_ to 0.0025 Hz and ∆f_2_ to 0.0005 Hz, substantially better than the best-performing PIDD² controller (∆f_1_ = 0.0103 Hz, ∆f_2_ = 0.0104 Hz) and significantly outperforming the PID controller (∆f_1_ = 0.2078 Hz, ∆f_2_ = 0.0486 Hz). Similarly, for tie-line power deviation (ΔP_tie_), the FPIDD²+PIDD²–GTO configuration achieves a near-negligible deviation of approximately 0.0001 p.u., demonstrating excellent inter-area power coordination. FPIDD²+PIDD² controller also excels in voltage response, where it provides the fastest and smoothest transient profile. When tuned with GTO, it achieves a settling time of approximately 1.8 s in both areas and registers the lowest voltage overshoots, reaching 0.1786 p.u. in Area 1 and 0.1913 p.u. in Area 2. These results underscore the FPIDD²+PIDD² controller’s effectiveness across multiple performance metrics, confirming its advantage over traditional control strategies.

The Integral Time Absolute Error (ITAE) values for each of the three control schemes, tuned using the three optimization algorithms, are presented in Table 15.

**Table 15:** The optimism integral time absolute error (ITAE) for all controllers.

Controller	Optimization Algorithm			Best ITAE	
	PSO	GTO	MPA	Value	Algorithm
PID	1060.23	858.83	599.90	599.90	MPA
PIDD²	535.35	534.59	535.78	534.59	GTO
FPIDD²+ PIDD²	66.09	52.18	54.76	52.18	GTO

The results presented in Table 15 confirm that the proposed FPIDD²+PIDD² controller offers a significant enhancement in system performance compared to conventional PID and PIDD² controllers. This is particularly evident in terms of the Integral of ITAE metric, where the FPIDD²+PIDD² controller, when optimized using the GTO, achieves the lowest ITAE value of 52.18. This exceptionally low value reflects the controller’s enhanced damping capability and its effectiveness in minimizing errors rapidly. In comparison, the best-performing configurations of PID and PIDD² controllers yield considerably higher ITAE values of 599.90 and 534.59, respectively, indicating slower dynamic responses and less effective error suppression.

Among the three metaheuristic optimization methods evaluated, MPA and GTO emerge as the most effective in fine-tuning controllers in most cases. They consistently produce the fastest, most stable transient responses, with minimal frequency and voltage deviations, further underscoring their suitability for optimizing advanced control structures in load frequency control applications.

#### Results summary

To further assess controller resilience under more complex conditions, this case study introduces both a photovoltaic (PV) unit in the first area at 20 s and a wind turbine in second area at 70 s. Under these stressed conditions, the conventional PID controller faced significant challenges, as evidenced by prolonged settling times in both deviation profiles of system frequency and tie line power. The best recorded ITAE under PID control was approximately 600. The PIDD² controller delivered marked improvements, reducing the ITAE was by approximately 10.9% compared to the conventional PID controller. Further enhancements were achieved using a hybrid control strategy, employing FPIDD² for LFC and PIDD² for AVR. This configuration delivered the most robust performance, characterized by minimal overshoot, and negligible steady-state error. Compared to the PID controller, ITAE was reduced by 91% and by 90% relative to PIDD² controller. Regarding optimization algorithms, when applied to PID controllers, the MPA achieved the best ITAE performance, followed closely by the GTO, then PSO, which was nearly 177% higher than that of MPA. However, for the PIDD² configuration, all three optimization algorithms yielded nearly identical results. A different pattern was observed for the FPIDD²+PIDD² hybrid setup, where GTO and MPA achieved comparable performance, while PSO resulted in an ITAE approximately 20% higher.

In summary, these results underscore a clear trend of progressive performance improvement, highlighting the superior control capabilities of the hybrid FPIDD²+PIDD². Notably, while optimization algorithms influence controller performance, particularly for simpler architectures like PID, their relative impact diminishes as more advanced controllers are employed. This suggests that controller architecture plays a more dominant role in determining overall system robustness. One plausible explanation is that advanced controllers such as PIDD² and FPIDD² incorporate additional degrees of freedom and adaptive features, which inherently enhance their ability to handle disturbances and parameter variations. As a result, they become less sensitive to fine-tuning by optimization algorithms, thereby narrowing the performance gap between optimizers. These findings imply that investing in better controller design may yield more substantial performance gains than focusing on optimization techniques.

### Case study no. 3: sensitivity analysis

#### System description

This case study is exactly the same as the pervious case study (case study No.2), but with some changes in parameters setting of power system, as shown per Table 16.

**Table 16:** Modified power system settings.

Model Constant	Model Value	Model Constant	Model Value	Model Constant	Model Value	Model Constant	Model Value
T_t_	0.15 s	K_ps2_	100	K_n_, K_e_	1.5	T_n_, T_e_	0.6, 1.5
T_gh_	0.12 s	T_12_	0.0867 MW	T_ps2_	10 s	R_g_, R_Th_, R_hyd_	1.2 Hz/MW

#### Simulation results

**Fig. 16:**
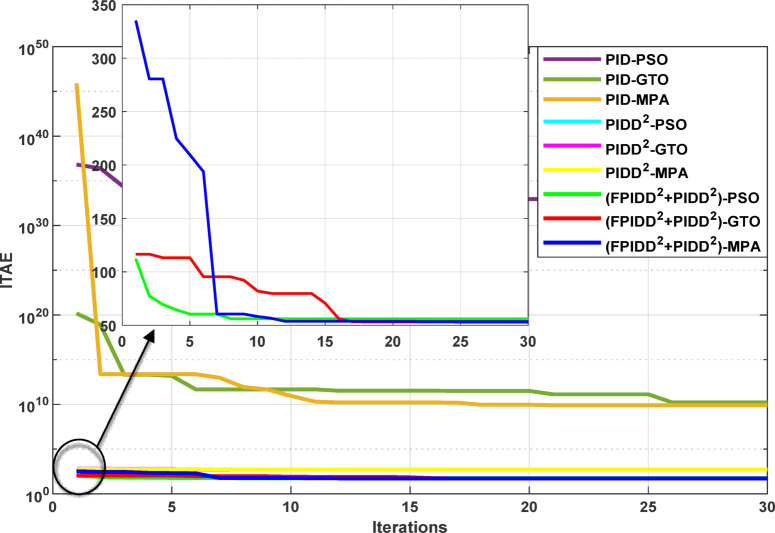
Convergence behavior of the ITAE during controller optimization in Case Study No. 3. The curves correspond to: PID–PSO (Purple), PID–GTO (Olive Green), PID–MPA (Light Brown), PIDD²–PSO (Cyan), PIDD²–GTO (Magenta), PIDD²–MPA (Yellow), (FPIDD²+PIDD²)–PSO (Light Green), (FPIDD²+PIDD²)–GTO (Red), and (FPIDD²+PIDD²)–MPA (Blue).

**Fig. 17:**
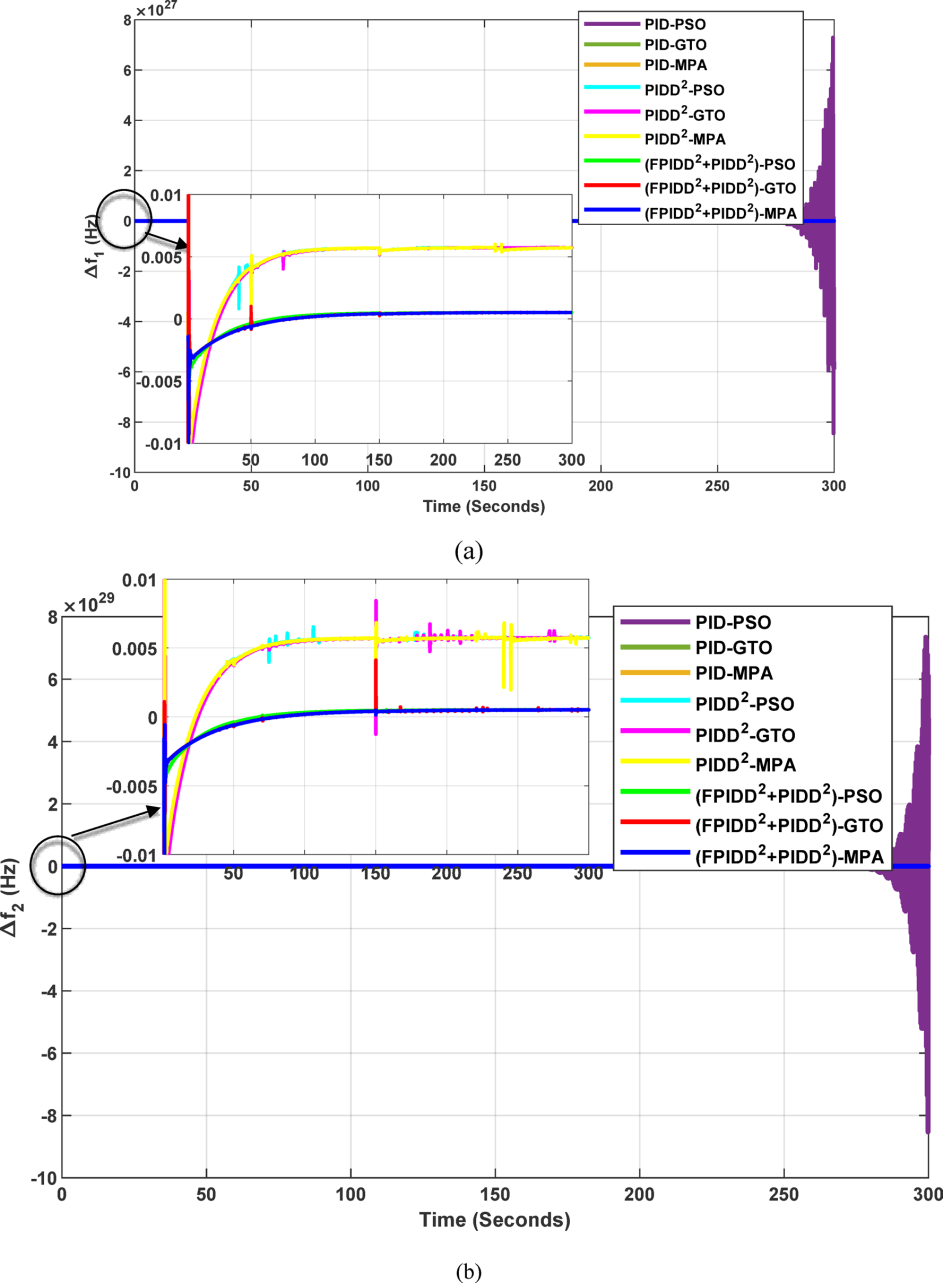

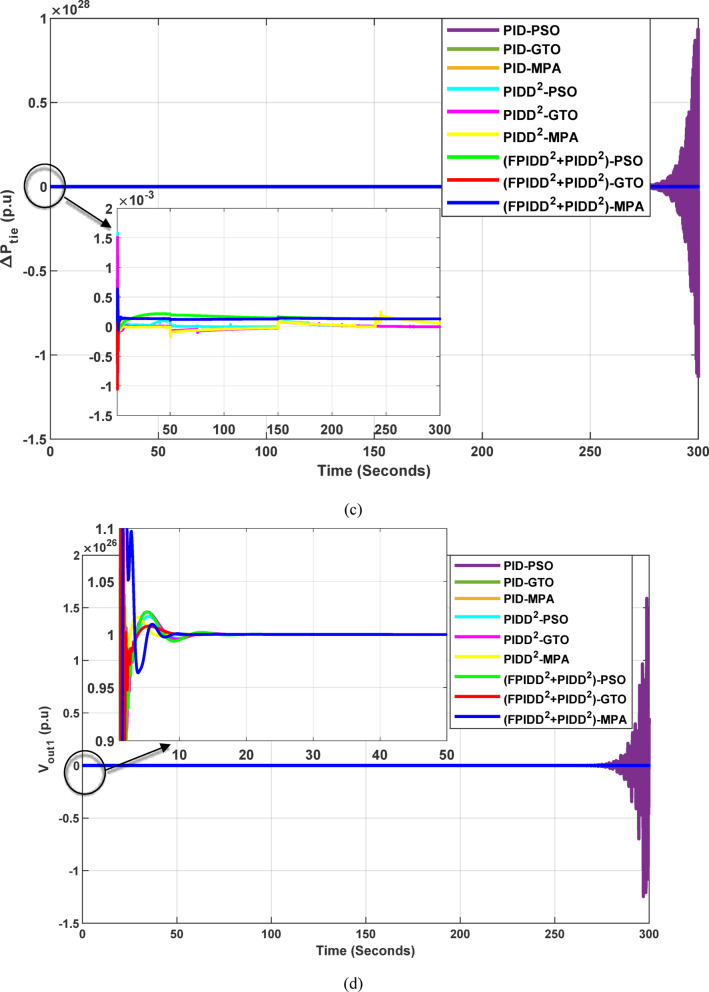

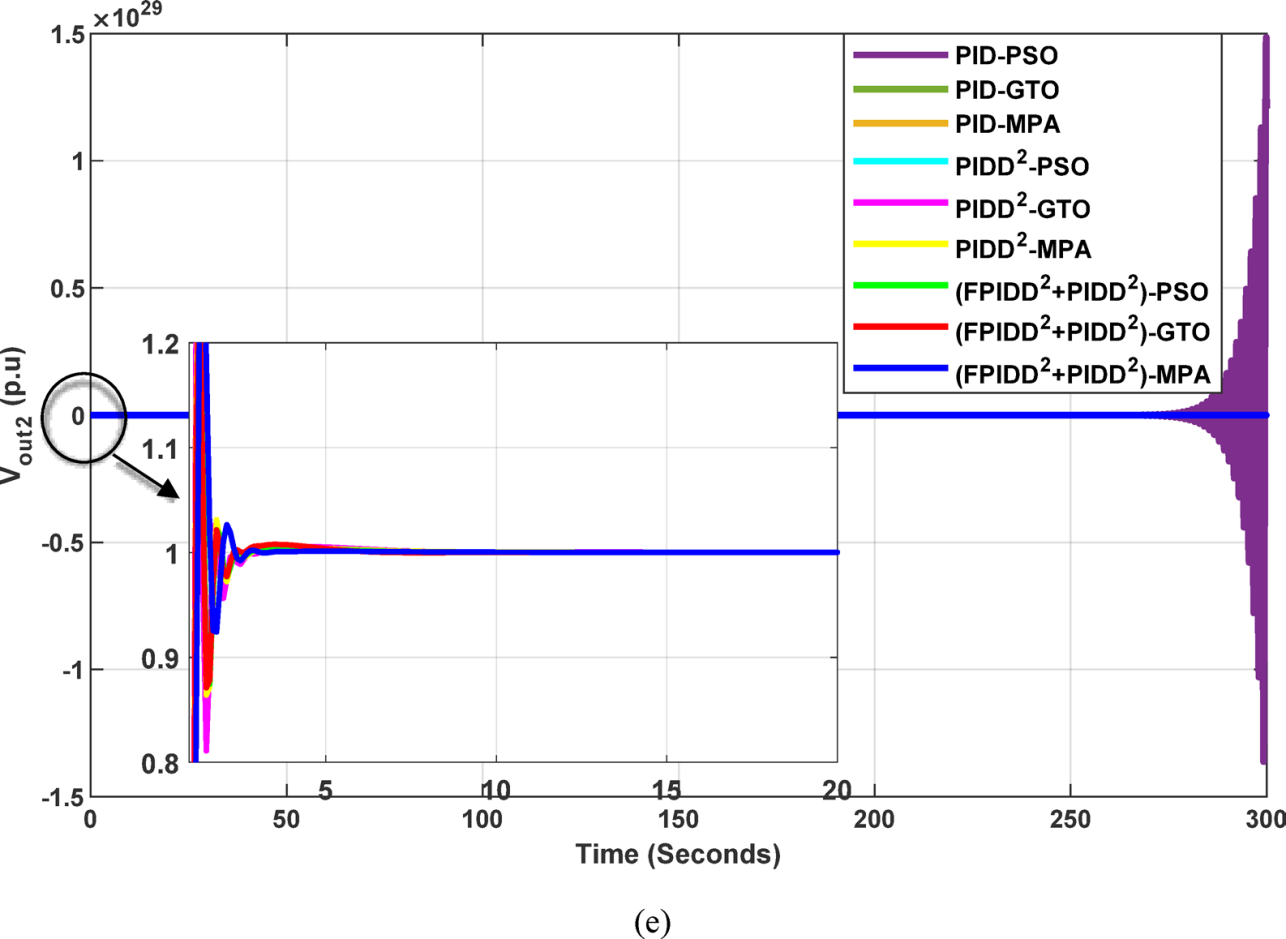
Dynamic responses of the two-area system of Case Study No.3: **a** ∆f₁, **b** ∆f₂, **c** ΔP_tie_, **d** V_out1_, **e** V_out2_. The curves correspond to: PID–PSO (Purple), PID–GTO (Olive Green), PID–MPA (Light Brown), PIDD²–PSO (Cyan), PIDD²–GTO (Magenta), PIDD²–MPA (Yellow), (FPIDD²+PIDD²)–PSO (Light Green), (FPIDD²+PIDD²)–GTO (Red), and (FPIDD²+PIDD²)–MPA (Blue). The conventional PID controller responses were omitted from the zoomed-in view to improve clarity, as they exhibit sever oscillations.

The convergence curves of the Integral Time Absolute Error (ITAE) are shown along with Time Absolute Error trends are illustrated in Fig. 16, while the power system dynamic responses are presented in Fig. 17.

Figure 16 shows that the proposed hybrid FPIDD²+PIDD² controllers demonstrate the most efficient convergence behavior. Their ITAE values drop sharply to near-zero within the early iterations, reflecting enhanced robustness and stability in parameter tuning. In comparison, the PIDD²-based controllers exhibit moderately improved convergence characteristics over conventional PID controllers, achieving lower ITAE values overall. Conversely, the conventional PID-based controllers exhibit extremely high ITAE values across the optimization process, reflecting slower convergence rates and limited optimization capability.

As depicted in Fig. 16, the classical PID controllers applied to the Load Frequency Control (LFC) loops in both Area-1 and Area-2 exhibit clear signs of instability. The frequency deviations ∆f_1_​ and ∆f_2_​ experience significant and sustained oscillations, indicating the limitations of traditional PID structures in maintaining frequency stability, particularly under the influence of system disturbances and nonlinear dynamics. In contrast, the PIDD²-based controllers provide notable improvements. These controllers effectively suppress oscillations and demonstrate enhanced damping characteristics, leading to a more stable frequency response compared to their classical counterparts. The most superior performance is delivered by the proposed hybrid FPIDD²+PIDD² control configurations. These advanced controllers maintain frequency deviations near zero throughout the entire simulation period, exhibiting excellent dynamic regulation and confirming their robustness and resilience under varying system conditions.

In terms of tie-line power deviation (ΔP_tie_​), the classical PID controllers again perform poorly, displaying significant oscillations that reflect an inability to maintain power exchange equilibrium between interconnected areas. The PIDD² controllers show moderate improvement, reducing the amplitude and persistence of the oscillations. However, the FPIDD²+PIDD² controllers clearly outperform both groups, maintaining near-zero tie-line power deviations and demonstrating strong disturbance rejection and sustained optimal inter-area power exchange. This underscores their effectiveness in preserving tie-line power stability in interconnected power systems.

Regarding the dynamic behavior of the Automatic Voltage Regulator (AVR) output voltages V_out1_​ and V_out2_​, the conventional PID-based controllers exhibit large-amplitude oscillations and instability, especially toward the end of the simulation. This indicates poor transient performance and limited robustness in voltage regulation. The PIDD²-based controllers improve voltage stability to some extent, offering smoother responses characterized by zero steady-state error, and enhanced damping. The hybrid FPIDD²+PIDD² controllers show the most stable and well-damped response among all configurations, maintain an almost constant voltage level throughout the simulation with negligible deviation, indicating excellent damping, rapid convergence, and high robustness.

In this case study, the optimal controllers’ sittings obtained for case study No.3 are shown per Tables 17, and 18, while the power system dynamic responses are presented in Table 19.

**Table 17:** Optimal controllers’ settings for the LFC loop in the first and second area.

Controller	Optim Algorithm	$\:{LFC|}_{Area1}$								$\:{LFC|}_{Area2}$							
		K_*P*_	K_I_	K_D1_	*N*_1_	K_D2_	*N*_2_	K_E_	K_CE_	K_*P*_	K_I_	K_D1_	*N*_1_	K_D2_	*N*_2_	K_E_	K_CE_
PID	PSO	18.5	2.0	2.0	1.1	**-**	**-**	**-**	**-**	92.9	1.3	1.8	1.5	**-**	**-**	**-**	**-**
PID	GTO	0.2	0.2	0.2	0.1	**-**	**-**	**-**	**-**	3.2	1.3	0.2	0.1	**-**	**-**	**-**	**-**
PID	MPA	0.1	1.7	1.9	0.2	**-**	**-**	**-**	**-**	2.9	0.1	2.0	0.2	**-**	**-**	**-**	**-**
PIDD²	PSO	100.0	2.0	2.0	2.0	10.0	100.0	**-**	**-**	100.0	2.0	0.8	2.0	10.0	100.0	**-**	**-**
PIDD²	GTO	100.0	2.0	1.2	1.6	10.0	100.0	**-**	**-**	100.0	2.0	2.0	1.4	1.1	98.1	**-**	**-**
PIDD²	MPA	100.0	2.0	2.0	2.0	4.2	96.1	**-**	**-**	100.0	2.0	2.0	1.8	3.2	94.8	**-**	**-**
FPIDD²+PIDD²	PSO	65.5	2.0	2.0	1.8	10.0	100.0	2.0	0.1	91.6	2.0	1.6	2.0	7.7	54.5	1.9	0.1
FPIDD²+PIDD²	GTO	100.0	2.0	0.1	2.0	1.3	100.0	2.0	0.1	100.0	2.0	2.0	2.0	0.1	100.0	2.0	0.1
FPIDD²+PIDD²	MPA	100.0	2.0	2.0	2.0	8.4	47.0	2.0	0.1	99.5	2.0	2.0	1.7	4.8	98.2	2.0	0.1

**Table 18:** Optimal controllers’ settings for the AVR loop in Area-1 and Area-2.

Controller	Optim Algorithm	$\:{AVR|}_{Area1}$								$\:AVR{|}_{Area2}$							
		K_*P*_	K_I_	K_D1_	*N*_1_	K_D2_	*N*_2_	K_E_	K_CE_	K_*P*_	K_I_	K_D1_	*N*_1_	K_D2_	*N*_2_	K_E_	K_CE_
PID	PSO	1.5	1.0	1.9	1.3	-	-	-	-	1.1	2.0	0.8	1.1	-	-	-	-
PID	GTO	0.2	0.1	0.1	0.1	-	-	-	-	0.1	0.1	0.1	0.1	-	-	-	-
PID	MPA	0.1	0.5	0.1	0.2	-	-	-	-	0.1	0.6	0.1	0.1	-	-	-	-
PIDD²	PSO	2.0	2.0	1.5	2.0	2.0	100.0	-	-	2.0	2.0	1.7	2.0	2.0	100.0	-	-
PIDD²	GTO	1.2	2.0	2.0	1.9	1.2	65.5	-	-	0.8	1.6	0.6	2.0	2.0	98.4	-	-
PIDD²	MPA	2.0	2.0	0.8	2.0	2.0	100.0	-	-	2.0	2.0	2.0	2.0	1.7	95.1	-	-
FPIDD²+PIDD²	PSO	2.0	2.0	1.5	1.6	2.0	100.0	-	-	2.0	1.8	1.2	1.7	2.0	100.0	-	-
FPIDD²+PIDD²	GTO	2.0	2.0	2.0	2.0	1.1	96.2	-	-	0.1	0.1	2.0	1.3	1.7	100.0	-	-
FPIDD²+PIDD²	MPA	0.7	2.0	2.0	0.2	1.4	0.1	-	-	2.0	2.0	0.9	1.5	1.0	60.5	-	-

**Table 19:** Dynamic responses of the system.

Controller	Optim. Algorithm	∆f_1_		∆f_2_		∆*P*_tie_		V_out1_			V_out2_		
		MO_∆f1_ (Hz)	MU_∆f1_ (Hz)	MO_∆f2_ (Hz)	MU_∆f2_ (Hz)	MO_∆*P*_ (*p*.u)	MU_∆*P*_ (*p*.u)	MP_− V1_ (*p*.u)	T_*r*−V1_ (Sec.)	T_s−V1_ (Sec.)	MP_− V2_ (*p*.u)	T_*r*−V2_ (Sec.)	T_s−V2_ (Sec.)
PID	PSO	N.A.	N.A.	N.A.	N.A.	N.A.	N.A.	N.A.	N.A.	N.A.	N.A.	N.A.	N.A.
PID	GTO	N.A.	N.A.	N.A.	N.A.	N.A.	N.A.	N.A.	N.A.	N.A.	N.A.	N.A.	N.A.
PID	MPA	N.A.	N.A.	N.A.	N.A.	N.A.	N.A.	N.A.	N.A.	N.A.	N.A.	N.A.	N.A.
PIDD²	PSO	0.006	−0.074	0.024	−0.100	0.002	−0.001	0.463	0.083	3.057	0.285	0.136	2.120
PIDD²	GTO	0.023	−0.103	0.065	−0.148	0.002	−0.001	0.482	0.109	5.722	0.286	0.136	2.122
PIDD²	MPA	0.026	−0.099	0.028	−0.100	0.001	−0.001	0.191	0.138	2.098	0.305	0.144	2.152
FPIDD²+PIDD²	PSO	0.001	−0.018	0.001	−0.020	0.000	0.000	0.451	0.084	5.583	0.292	0.147	2.135
FPIDD²+PIDD²	GTO	0.045	−0.054	0.004	−0.035	0.000	−0.001	0.437	0.110	2.659	0.274	0.146	2.123
FPIDD²+PIDD²	MPA	0.001	−0.010	0.001	−0.015	0.001	0.000	0.341	0.295	4.697	0.313	0.181	2.195

Table 19 clearly demonstrate that the proposed FPIDD²+PIDD² controller outperforms the conventional PIDD² controller across all optimization algorithms, while the PID controller fails to produce valid or stable responses (N.A.), confirming its inadequacy under the given operating conditions.

Among the evaluated schemes, the FPIDD²+PIDD² configuration achieves the best overall dynamic and steady-state performance. For instance, when optimized using the MPA algorithm, the controller yields the smallest frequency deviations, with the minimum overshoot and undershoot of approximately + 0.0006 Hz and − 0.01 Hz, respectively. Meanwhile, the PIDD² controller exhibits larger fluctuations up to ± 0.10 Hz, while the PID controller becomes unstable, resulting in rapidly growing oscillations. A similar trend is observed in the tie-line power deviations, where the (FPIDD²+PIDD²)–MPA configuration maintains minimal deviations of around 0.0006 p.u. Conversely, the PIDD² controller shows relatively higher deviations (≈ ± 0.0001 p.u.), and the PID controller again fails to ensure stability. In terms of voltage regulation, both of the PIDD^2^ and FPIDD²+PIDD² controllers exhibits faster settling and smoother transient behavior than the traditional PID controller, which. is unable to maintain voltage stability, leading to divergent oscillations in the AVR loop.

The Integral Time Absolute Error (ITAE) values for each of the three control schemes, tuned using the three optimization algorithms, are presented in Table 20.

**Table 20:** The optimism integral time absolute error (ITAE) for all controllers.

Controller	Optimization Algorithm			Best ITAE	
	PSO	GTO	MPA	Value	Algorithm
PID	> 10^9^	> 10^9^	> 10^9^	N.A.	N.A.
PIDD²	541.75	540.30	540.44	540.30	GTO
FPIDD²+ PIDD²	56.19	52.79	53.47	52.79	GTO

The results presented in Table 20 clearly highlight the superior performance of the proposed FPIDD²+PIDD² controller over the conventional PIDD² and PID controllers. The PID controller fails to maintain stable operation producing excessively large ITAE value (> 10⁹). In contrast, all configurations of the FPIDD²+PIDD² controller demonstrate substantial improvements in dynamic performance and error minimization. Notably, the (FPIDD²+PIDD²)–GTO configuration achieves the lowest ITAE value of 52.79. Meanwhile, the PIDD² controllers record a considerably higher ITAE value around 540, signifying slower system response and greater steady-state deviations. These results affirm that when the FPIDD²+PIDD² is coupled with advanced optimization techniques, it markedly enhances both transient and steady-state performance in interconnected LFC–AVR systems.

#### Results summary

Beyond renewable energy integration, this case study investigates controller performance under altered power system parameters to emulate real-world variability. The study modifies the time constants of key components, such as steam and hydro turbines, AVR exciters, generators, and the overall power network, while also incorporating nonlinearities and coupling effects typical of complex multi-area systems.

Under these challenging conditions, the classical PID controller demonstrated instability and unsatisfactory performance, yielding ITAE values exceeding billions. This outcome underscores the inherent limitations of PID control in handling nonlinear dynamics and strong inter-area coupling. In contrast, the PIDD² controller exhibited marked improvements, achieving reduced overshoot, faster rise times, and smaller steady-state errors, with a minimum ITAE of approximately 540.

Further advancements were realized through the hybrid FPIDD²+PIDD² control strategy. This configuration delivered the most stable and responsive performance across all test scenarios, achieving a 90.2% reduction in ITAE compared to the standalone PIDD² controller, along with superior damping, minimal overshoot, and strong steady-state precision.

Regarding optimization algorithms, applying the three optimization methods to PID controllers under the altered system conditions consistently produced extremely high ITAE values, reaffirming the inadequacy of the conventional PID structure under such variability. Conversely, when the same optimization algorithms were applied to the PIDD² and hybrid FPIDD²+PIDD² controllers, the outcomes were nearly identical within each control type, indicating that advanced control structures possess intrinsic robustness and adaptability, largely independent of the specific optimization technique employed.

### Case study no. 4: the effect random load perturbation

#### System description

**Fig. 18:**
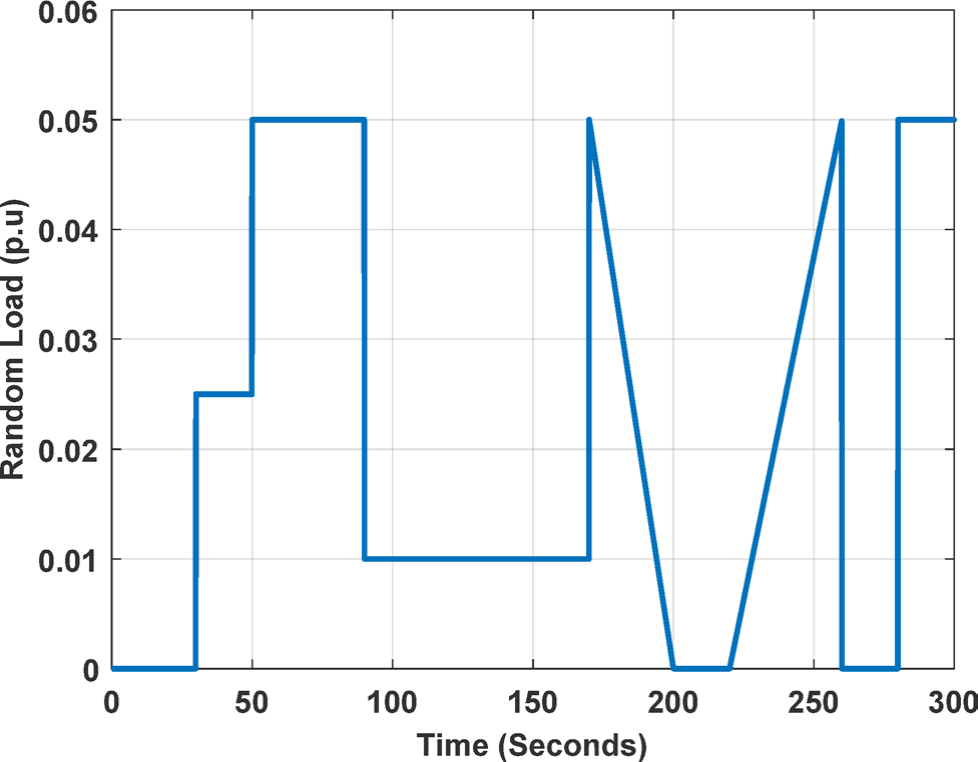
Random Load Perturbation (RLP) signal injected to Area-1 during Case Study No.4.

This case study is exactly the same as case study No.2, but Area-1 is subjected to a random load variation (RLP) as shown in Fig. 18.

#### Simulation results

**Fig. 19:**
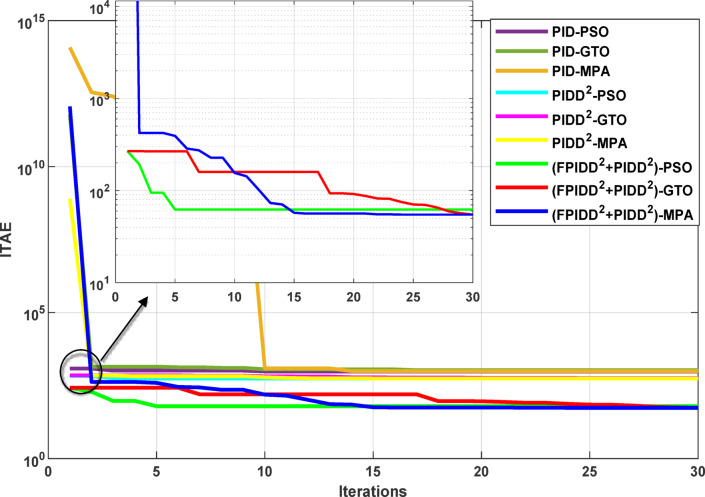
Convergence behavior of the ITAE during controller optimization in Case Study No. 4. The curves correspond to: PID–PSO (Purple), PID–GTO (Olive Green), PID–MPA (Light Brown), PIDD²–PSO (Cyan), PIDD²–GTO (Magenta), PIDD²–MPA (Yellow), (FPIDD²+PIDD²)–PSO (Light Green), (FPIDD²+PIDD²)–GTO (Red), and (FPIDD²+PIDD²)–MPA (Blue).

**Fig. 20:**
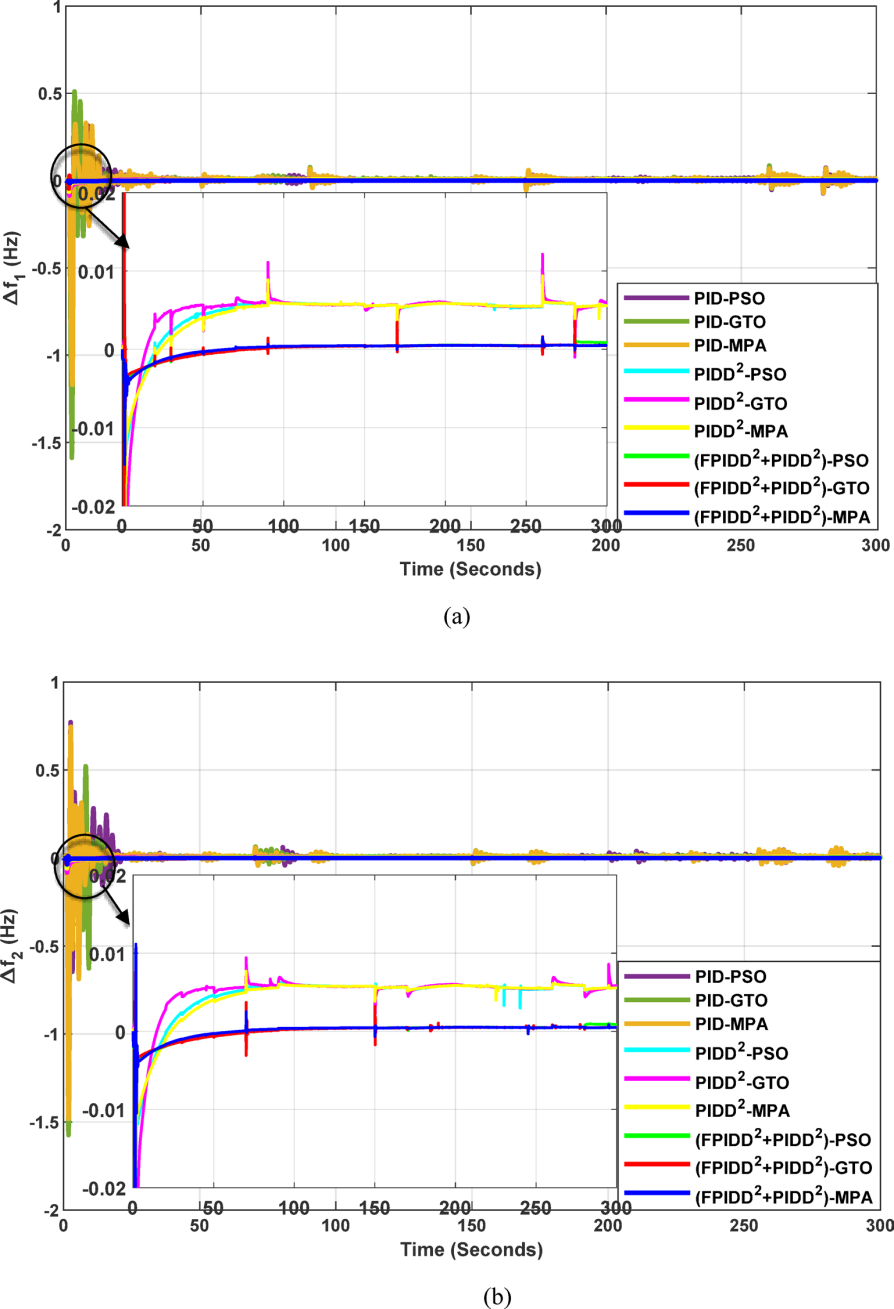

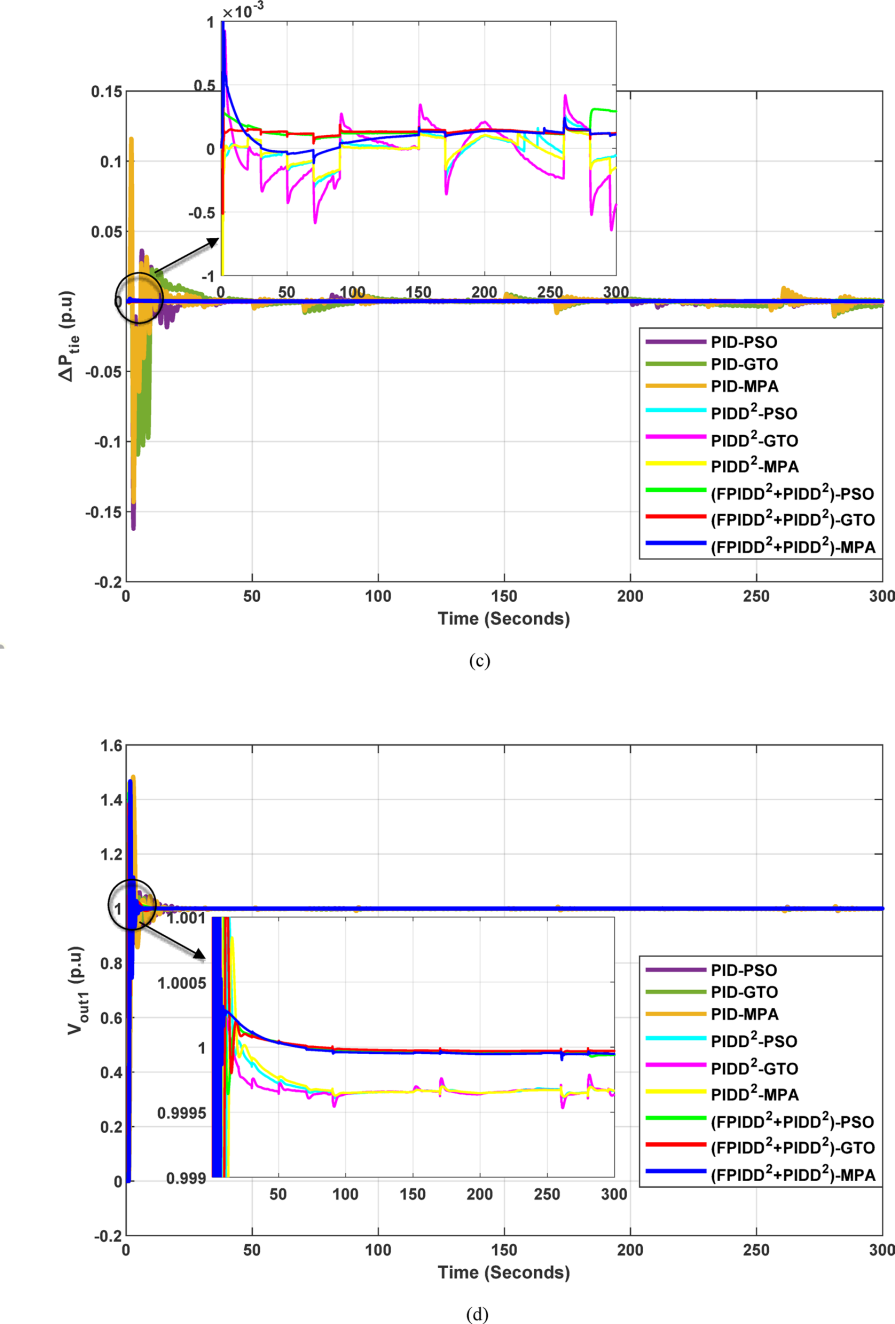

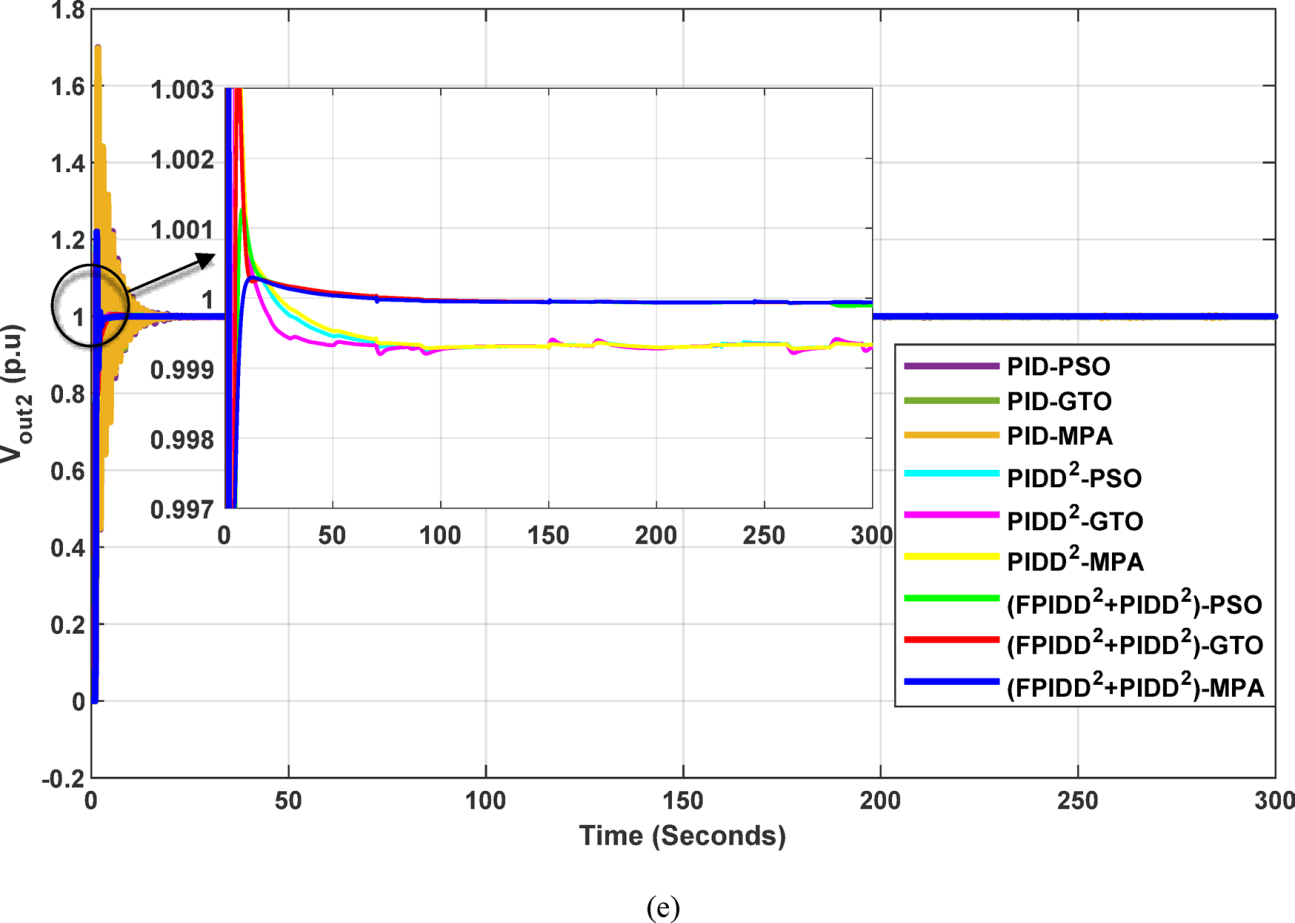
Dynamic responses of the two-area system of Case Study No.4: **a** ∆f₁, **b** ∆f₂, **c** ΔP_tie_, **d** V_out1_, **e** V_out2_. The curves correspond to: PID–PSO (Purple), PID–GTO (Olive Green), PID–MPA (Light Brown), PIDD²–PSO (Cyan), PIDD²–GTO (Magenta), PIDD²–MPA (Yellow), (FPIDD²+PIDD²)–PSO (Light Green), (FPIDD²+PIDD²)–GTO (Red), and (FPIDD²+PIDD²)–MPA (Blue). The conventional PID controller responses were omitted from the zoomed in view to improve clarity, as they exhibit notable fluctuations.

The convergence curves of the Integral Time Absolute Error (ITAE) are shown along with Time Absolute Error trends are illustrated in Fig. 19, while the power system dynamic responses are presented in Fig. 20.

As illustrated in Fig. 19, most controller configurations exhibit a rapid decrease in ITAE values during the initial iterations, indicating efficient early-stage learning and convergence behavior. However, conventional PID-based schemes tend to plateau at relatively high ITAE levels, reflecting limited optimization accuracy and suboptimal transient performance. Meanwhile, the PIDD^2^-based controllers demonstrate moderate improvements in ITAE reduction, yet they remain less effective in achieving optimal tuning and convergence compared to the proposed hybrid approach.

In contrast, the FPIDD^2^ + PIDD^2^ control configuration shows markedly superior convergence characteristics, achieving the lowest final ITAE values along with the fastest stabilization rate. This performance indicates a more robust global search capability, effectively avoiding premature convergence and enabling the identification of near-optimal controller gains delivering enhanced dynamic response and improving steady-state performance.

Figure 20 clearly illustrates that the conventional PID controllers exhibit more pronounced transient oscillations and longer settling times. Notably, the PID controller shows significant oscillatory behavior, with high peak frequency deviations, indicating suboptimal damping characteristics and delayed system recovery. In contrast, the PIDD² controllers demonstrate enhanced dynamic performance, characterized by reduced overshoot and faster stabilization. However, minor oscillations with small-amplitude fluctuations persist throughout the simulation period. The most significant performance improvement is observed in the hybrid control configuration (PIDD²+PIDD²), particularly when optimized using the MPA algorithm. This configuration achieves the lowest frequency deviations and exhibits near-instantaneous convergence to zero deviation, consistently outperforming all other schemes. These results underscore the effectiveness of the PIDD²+PIDD² hybrid control strategy in minimizing frequency fluctuations, as it demonstrates negligible overshoot, rapid settling time, and robust oscillation suppression.

Regarding tie-line power deviation (ΔP_tie_), the results indicate that among the conventional PID-based designs, significant oscillations and the largest peak deviations were observed. The application of PIDD² controllers leads to noticeable improvements, including a moderate reduction in peak deviations and enhanced damping characteristics compared to conventional PID controllers. However, some fluctuations persist during the simulation. The most favorable performance is achieved with the hybrid control schemes combining FPIDD² and PIDD² controllers, demonstrating negligible overshoot, and minimal oscillations. This performance highlights its superior capability in maintaining inter-area tie-line power stability and its effectiveness in suppressing tie-line power deviations.

Regarding the output voltage response, the conventional PID controllers show higher overshoot and longer settling times, indicating less effective damping of system oscillations. In contrast PIDD^2^, display significantly improved transient responses with reduced overshoot and faster convergence to the steady-state value. Among the evaluated approaches, the combined controller (FPIDD^2^ + PIDD^2^), demonstrates the best overall performance, achieving rapid voltage stabilization with negligible overshoot and minimal steady-state error.

In summary, the overall results confirm the superior performance of the proposed FPIDD²+PIDD² hybrid controller compared to conventional PID and standalone PIDD² schemes, as it effectively suppresses both frequency and tie-line power deviations while ensuring fast and stable voltage recovery, demonstrating its robustness and efficiency in enhancing overall system stability and control performance.

In this case, the optimal controllers’ sittings obtained for case study No.4 are shown per Table 21, and Table 22, while the power system dynamic responses are presented in Table 23.

**Table 21:** Optimal controllers’ settings for the LFC loop in the first and second area.

Controller	Optim. Algorithm	$\:{LFC|}_{Area1}$								$\:{LFC|}_{Area2}$							
		K_*P*_	K_I_	K_D1_	*N*_1_	K_D2_	*N*_2_	K_E_	K_CE_	K_*P*_	K_I_	K_D1_	*N*_1_	K_D2_	*N*_2_	K_E_	K_CE_
PID	PSO	0.1	1.4	1.3	0.5	**-**	**-**	**-**	**-**	0.2	1.3	1.3	0.2	-	-	-	-
PID	GTO	0.1	0.3	1.7	0.3	**-**	**-**	**-**	**-**	0.1	0.2	0.5	0.3	-	-	-	-
PID	MPA	0.1	1.2	1.2	0.5	**-**	**-**	**-**	**-**	0.1	1.4	1.1	0.2	-	-	-	-
PIDD²	PSO	81.5	2.0	2.0	2.0	9.7	67.7	**-**	**-**	80.5	2.0	1.7	1.5	7.9	83.1	-	-
PIDD²	GTO	40.0	2.0	1.8	1.3	9.2	61.1	**-**	**-**	34.3	2.0	2.0	1.1	9.1	68.8	-	-
PIDD²	MPA	99.8	2.0	2.0	1.5	10.0	51.2	**-**	**-**	100.0	2.0	2.0	2.0	10.0	49.0	-	-
FPIDD²+PIDD²	PSO	100.0	2.0	2.0	2.0	10.0	58.7	2.0	0.1	83.9	2.0	2.0	2.0	6.9	96.5	1.9	0.1
FPIDD²+PIDD²	GTO	100.0	2.0	0.1	2.0	10.0	100.0	2.0	0.0	100.0	2.0	2.0	2.0	10.0	0.1	2.0	0.1
FPIDD²+PIDD²	MPA	99.8	2.0	2.0	2.0	9.6	60.6	2.0	0.0	48.6	2.0	1.9	0.1	3.6	100.0	2.0	0.0

**Table 22:** Optimal controllers’ settings for the AVR loop in the first and second area.

Controller	Optimization Algorithm	$\:{AVR|}_{Area1}$								$\:AVR{|}_{Area2}$							
		K_*P*_	K_I_	K_D1_	*N*_1_	K_D2_	*N*_2_	K_E_	K_CE_	K_*P*_	K_I_	K_D1_	*N*_1_	K_D2_	*N*_2_	K_E_	K_CE_
PID	PSO	0.2	0.8	0.4	2.0	-	-	-	-	0.7	1.2	0.4	1.0	-	-	-	-
PID	GTO	0.8	0.8	0.3	0.4	-	-	-	-	0.5	0.6	0.6	0.7	-	-	-	-
PID	MPA	0.1	0.7	0.4	2.0	-	-	-	-	0.7	1.2	0.3	1.0	-	-	-	-
PIDD²	PSO	1.9	2.0	1.6	1.6	1.6	100.0	-	-	1.8	1.9	2.0	1.7	2.0	100.0	-	-
PIDD²	GTO	1.7	2.0	1.2	1.8	1.0	75.6	-	-	1.5	0.9	2.0	1.6	1.7	67.4	-	-
PIDD²	MPA	2.0	2.0	1.9	2.0	1.7	99.6	-	-	0.1	2.0	2.0	2.0	2.0	74.6	-	-
FPIDD²+PIDD²	PSO	1.9	1.8	2.0	2.0	1.1	100.0	-	-	1.0	2.0	2.0	2.0	2.0	100.0	-	-
FPIDD²+PIDD²	GTO	2.0	2.0	1.3	1.3	1.7	100.0	-	-	0.1	2.0	2.0	2.0	2.0	100.0	-	-
FPIDD²+PIDD²	MPA	1.9	1.2	2.0	0.1	0.4	0.2	-	-	0.7	1.9	0.2	0.7	0.9	21.4	-	-

**Table 23:** Dynamic responses of the system.

Controller	Optim Algorithm	∆f_1_		∆f_2_		∆*P*_tie_		V_out1_			V_out2_		
		MO_∆f1_ (Hz)	MU_∆f1_ (Hz)	MO_∆f2_ (Hz)	MU_∆f2_ (Hz)	MO_∆*P*_ (*p*.u)	MU_∆*P*_ (*p*.u)	MP_− V1_ (*p*.u)	T_*r*−V1_ (Sec.)	T_s−V1_ (Sec.)	MP_− V2_ (*p*.u)	T_*r*−V2_ (Sec.)	T_s−V2_ (Sec.)
PID	PSO	0.417	−1.276	0.772	−1.464	0.103	−0.162	0.471	0.531	12.073	0.699	0.252	13.652
PID	GTO	0.511	−1.588	0.522	−1.574	0.077	−0.130	0.380	0.397	8.483	0.674	0.254	11.746
PID	MPA	0.331	−1.170	0.746	−1.483	0.116	−0.143	0.483	0.610	12.364	0.697	0.252	12.830
PIDD²	PSO	0.010	−0.079	0.009	−0.078	0.001	−0.001	0.436	0.102	2.620	0.156	0.145	3.146
PIDD²	GTO	0.012	−0.086	0.009	−0.083	0.001	−0.001	0.230	0.151	2.146	0.196	0.152	2.906
PIDD²	MPA	0.012	−0.064	0.008	−0.059	0.001	−0.001	0.459	0.077	3.214	0.195	0.145	3.188
FPIDD²+PIDD²	PSO	0.002	−0.015	0.001	−0.016	0.000	0.000	0.423	0.111	2.971	0.183	0.170	3.009
FPIDD²+PIDD²	GTO	0.028	−0.046	0.008	−0.034	0.000	−0.001	0.384	0.112	4.806	0.164	0.144	3.272
FPIDD²+PIDD²	MPA	0.002	−0.015	0.011	−0.036	0.002	0.000	0.467	0.246	4.135	0.220	0.200	2.589

As presented in Table 23, the performance of the three controller configurations demonstrates the progressive enhancement in system stability and dynamic response.

In terms of frequency regulation, the conventional PID controllers exhibit the weakest performance, characterized by pronounced oscillations with overshoot and undershoot values reaching up to ± 1.5 Hz. This behavior reflects poor damping capability and slow recovery following load disturbances. The PIDD² controllers significantly enhance frequency stability by reducing both the magnitude and duration of oscillations, with the worst-case overshoot and undershoot limited to approximately 0.01 Hz and − 0.06 Hz, respectively. The hybrid FPIDD²+PIDD² controller achieves the best performance, maintaining almost negligible oscillations, with overshoot and undershoot values as low as 0.002 Hz and − 0.015 Hz, indicating exceptional damping and rapid convergence to steady state.

Regarding tie-line power deviation (ΔP_tie_), the PID controllers display the largest fluctuations, reaching up to 0.12 p.u., signifying weak inter-area coordination and delayed oscillation suppression. The PIDD² controllers markedly improve tie-line power stability, limiting deviations to around ± 0.001 p.u., although minor transient fluctuations remain. In contrast, the hybrid FPIDD²+PIDD² configuration demonstrates the most stable tie-line power behavior, maintaining minimal deviations and effectively suppressing inter-area oscillations.

In terms of voltage response, the PID controllers again show inferior performance, with overshoots reaching approximately 0.7 p.u. in some cases and extended settling times, indicating inadequate voltage regulation and weak transient damping. The PIDD² controllers yield notable improvement, achieving faster settling and smaller overshoot values of about 0.20 p.u., thereby enhancing voltage stability. The hybrid FPIDD²+PIDD² controller outperforms both configurations, delivering rapid voltage stabilization with minimal steady-state error. In summary, across all three performance indicators, frequency, tie-line power, and voltage, the hybrid FPIDD²+PIDD² controller consistently outperforms both the PIDD² and conventional PID configurations, delivering superior damping, faster recovery, and enhanced robustness against dynamic disturbances.

Integral Time Absolute Error (ITAE) values for each of the three control schemes, tuned using the three optimization algorithms, are presented in Table 24.

**Table 24:** The optimism integral time absolute error (ITAE) for all controllers.

Controller	Optimization Algorithm			Best ITAE	
	PSO	GTO	MPA	Value	Algorithm
PID	965.45	1066.06	993.59	993.59	PSO
PIDD²	553.15	568.51	551.81	551.81	MPA
FPIDD²+ PIDD²	62.19	54.85	54.84	54.84	MPA

The results presented in Table 24 demonstrate the substantial performance improvement achieved by the proposed FPIDD^2^ + PIDD^2^ controller over both classical PID and fractional-order PIDD^2^ controllers. This hybrid control scheme achieved the lowest ITAE value of 54.84, indicating highly efficient error minimization and enhanced transient response. In comparison, the conventional PID controller recorded the highest ITAE, approaching 1000, reflecting poor dynamic performance and limited control accuracy. The PIDD^2^ controller showed moderate improvement, reducing the ITAE to approximately 550, almost half of the PID value, yet it still falls short of the performance achieved by the hybrid configuration. These findings confirm that FPIDD^2^ + PIDD^2^ significantly enhances control precision, system stability, and damping effectiveness, thereby validating the robustness and efficiency of the proposed control strategy.

#### Results summary

The final case study introduces a random load perturbation in Area-1, serving as a stress test to evaluate the robustness of the controller under severe dynamic disturbances. Under these challenging conditions, the conventional PID controller faced significant limitations, with the best recorded ITAE reaching approximately 965. In contrast, the PIDD² controller showed substantial improvements across all dynamic performance indicators. It effectively controlled frequency deviations, tie-line power, and terminal voltage, reducing the ITAE to about half of the PID controller’s value, with a final ITAE of around 550.

The hybrid control strategy (FPIDD² +PIDD²), delivered the most impressive results overall. This configuration demonstrated minimal steady-state error and reduced overshoot, further lowering the ITAE by 90.1% compared to the standalone PIDD² controller, and by 94.3% compared to the PID controller. These results underscore the superior resilience, precision, and dynamic performance of the hybrid FPIDD²+PIDD² approach in managing large-scale load disturbances in interconnected power systems.

Additionally, it was noted that all optimization algorithms produced nearly identical ITAE values, showing only minor differences within each control technique (PID, PIDD², and FPIDD²+PIDD). This further emphasizes that, under severe disturbance scenarios, the choice of controller architecture has a far more significant impact on system stability and performance than the specific optimizer used. A plausible explanation is that advanced controller architectures inherently exhibit greater adaptability features that are crucial for responding to unpredictable conditions such as RLP. Consequently, these findings suggest a strategic shift in focus: rather than relying primarily on sophisticated optimization techniques for conventional controllers, greater performance gains may be realized by developing more advanced control architectures.

#### Statistical performance evaluation

The computational effort also was quantified using the average execution time obtained through MATLAB/Simulink profiling on an Intel^®^ Core™ i5-4300U processor (@ 1.90 GHz 2.50 GHz, 12 GB RAM). The results are summarized as shown per Table 25, which provides a comprehensive summary of the simulation results obtained from the four case studies employed to evaluate the three control strategies, including conventional PID, PIDD², and the proposed hybrid FPIDD²+PIDD² controllers, with each optimally tuned using the three metaheuristic optimization algorithms PSO, GTO, and MPA.

**Table 25:** Computational complexity.

Controller	Average execution time per 1 control cycle (minutes)	Relative computational cost (%)	Remarks
PID	13	100	Baseline reference
PIDD²	16	123	Slight increase due to extra derivative term
FPIDD²+PIDD²	22	169	Moderate cost; feasible for real-time deployment

Table 26 presents the comparative performance of PID, PIDD², and the proposed FPIDD²+PIDD² hybrid controller across four case studies, with performance evaluated using the ITAE criterion and controller parameters optimized via PSO, GTO, and MPA. The results demonstrate that the FPIDD²+PIDD² hybrid controller outperforms conventional PID and standalone PIDD² schemes in all cases, achieving notable improvements in control performance. The overall uncertainty in the results is estimated at approximately 5%, which remains within acceptable limits for control system simulations and experimental validations, and does not compromise the validity of the comparative analysis or the conclusions drawn.

**Table 26:** Integral time absolute error (ITAE) for all control schemes across all four case studies.

Case Study No.	Control scheme	ITAE based on			Statistical analysis				ITAE reduction of FPIDD²+ PIDD² compared to	
					Mean (µ)	Standard deviation (σ)	Variance (σ^2^)	Relative Uncertainty (σ/µ × 100%)		
		PSO	GTO	MPA					PID	PIDD²
1 st case study	PID	585.2	528.8	550.2	554.7	23.3	540.7	4.3%	90.3%	90.2%
	PIDD²	549.3	549.4	548.0	548.9	0.6	0.4	0.1%		
	FPIDD²+ PIDD²	53.6	53.9	53.2	53.6	0.3	0.1	0.6%		
2nd case study	PID	1060.2	858.8	599.9	839.7	188.4	35501.2	24.2%	93.1%	89.2%
	PIDD²	535.4	534.6	535.8	535.2	0.5	0.2	0.1%		
	FPIDD²+ PIDD²	66.1	52.2	54.8	57.7	6	36.5	10.7%		
3rd case study	PID	> 10^9^	> 10^9^	> 10^9^	N.A.	N.A.	N.A.	N.A.	100.0%	90.0%
	PIDD²	541.8	540.3	540.4	540.8	0.7	0.4	0.1%		
	FPIDD²+ PIDD²	56.2	52.8	53.5	54.2	1.5	2.2	2.8%		
4th case study	PID	965.5	1066.1	993.6	1008.4	42.4	1796.2	4.2%	94.3%	89.7%
	PIDD²	553.2	568.5	551.8	557.8	7.6	57.4	1.4%		
	FPIDD²+ PIDD²	62.2	54.9	54.8	57.3	3.5	12	6.1%		
Average								5.0%	93.9%	90.2%

Tables 27 and 28 present the comparative percentage improvements in the dynamic response parameters of the proposed hybrid (FPIDD²+PIDD²) controllers over PID and PIDD^2^ controllers grouped by each one of the three optimization techniques. The results highlight the capability of the hybrid controller in enhancing system stability and dynamic response across various performance indices.

**Table 27:** Percentage improvement in dynamic response parameters of hybrid (FPIDD²+PIDD²) controllers compared to the PID Controller.

Controller	∆f_1_ (Hz)		∆f_2_ (Hz)		∆*P*_tie_ (*p*.u)		V_out1_ (*p*.u)			V_out2_ (*p*.u)		
	MO_∆f1_	MU_∆f1_	MO_∆f2_	MU_∆f2_	MO_∆*P*_	MU_∆*P*_	MP_− V1_	T_*r*−V1_	T_s−V1_	MP_− V2_	T_*r*−V2_	T_s−V2_
PSO	97%	91%	99%	91%	98%	100%	43%	85%	38%	71%	68%	71%
GTO	−25%	56%	70%	69%	74%	49%	25%	36%	49%	48%	29%	48%
MPA	71%	78%	69%	74%	46%	73%	23%	−17%	53%	49%	12%	59%
Average	48%	75%	79%	78%	73%	74%	30%	35%	46%	56%	36%	59%

The results of case study # 3 have been omitted for simplicity.

**Table 28:** Percentage improvement in dynamic response parameters of hybrid (FPIDD²+PIDD²) controllers compared to the PIDD^2^ Controller.

Controller	∆f_1_ (Hz)		∆f_2_ (Hz)		∆*P*_tie_ (*p*.u)		V_out1_ (*p*.u)			V_out2_ (*p*.u)		
	MO_∆f1_	MU_∆f1_	MO_∆f2_	MU_∆f2_	MO_∆*P*_	MU_∆*P*_	MP_− V1_	T_*r*−V1_	T_s−V1_	MP_− V2_	T_*r*−V2_	T_s−V2_
PSO	43%	72%	91%	76%	36%	75%	31%	23%	−73%	5%	1%	20%
GTO	−3%	45%	67%	64%	50%	13%	8%	21%	−14%	20%	3%	0%
MPA	94%	79%	58%	62%	−150%	91%	12%	−115%	−8%	4%	−22%	0%
Average	45%	65%	72%	67%	−21%	60%	17%	−24%	−32%	10%	−6%	7%

## Conclusion

This study investigates the performance of a hybrid FPIDD²+PIDD² control strategy for coordinated load-frequency control (LFC) and automatic-voltage regulation (AVR) in complex power-system environments. Comparative analyses carried out across four progressively challenging case studies demonstrate that the proposed hybrid scheme offers superior robustness and adaptability compared with the conventional PID controller and the improved PIDD² controller under the tested conditions. The classical PID controller serves as a useful baseline but shows clear limitations such as higher overshoot, longer settling times, increased steady-state error, and occasionally oscillation or instability when confronted with system nonlinearities, inter-area coupling and dynamic uncertainties. The PIDD² controller delivers measurable improvement over the PID. Most significantly, the hybrid FPIDD²+PIDD² configuration attains the best overall performance in the considered scenarios by reducing the integral time-weighted absolute error (ITAE) by up to approximately 94% relative to the PID controller and by about 90% relative to the PIDD² controller. In addition, the study explores the influence of tuning-optimizer choice (PSO, MPA and GTO) on controller performance. Optimizer selection markedly affects the PID controller’s performance whereas its impact on the PIDD² and hybrid FPIDD²+PIDD² controllers is far less pronounced. This finding suggests that the controller architecture exerts a stronger influence on system behavior than the specific optimization method.

Although this study found that the hybrid FPIDD²+PIDD² controller demonstrated favorable dynamic characteristics and markedly improved performance across most indices, the hybrid control scheme did not outperform the PIDD² controller on all dynamic metrics which suggests potential for further refinement via offline optimization and adaptive fuzzy scaling.

In addition. The analysis was carried out under idealized system conditions with parameter variations modelled deterministically. Real-world factors such as communication delays, measurement noise, actuator nonlinearities and parameter drift were not explicitly represented. Practical implementation may thus face challenges in parameter tuning, noise sensitivity and computational load. Moreover, the real-time demands of fuzzy inference and second-derivative filtering could require hardware optimization for embedded implementation. Accordingly, future work may extend and validate the proposed FPIDD²+PIDD² control framework in larger, more complex multi-area power systems that integrate renewables. This will include real-time evaluation via hardware-in-the-loop experiments and development of adaptive online re-tuning strategies to enhance long-term robustness. Further research should establish systematic methodologies for configuring the FPIDD² controller, defining appropriate upper and lower bounds for tuning parameters, population size, iterations number, fuzzy rule-bases, membership functions and crisp ranges. To support scalability, comparability and wider adoption of intelligent control architectures, it is also recommended to develop standardized benchmarks, performance metrics and test cases.

## Data Availability

The datasets used and/or analyzed during the current study available from the corresponding author on reasonable request.

## List of symbols

∆f_1_: Area (1) frequency deviation (Hz)
∆f_2_: Area (2) frequency deviation (Hz)
∆P_tie_: The power deviation of the tie-line (p.u.)
AVR: Automatic Voltage Regulator
B_1_, B_2_: Frequency bias coefficients
B_g_: Valve positioner time constant
C_12_: Synchronization coefficient
CE: Rate of fuzzy controller error change
C_g_: Gas turbine valve positioner
CL: Control law
COG: Center of gravity
E: Error of fuzzy controller
E_ss_: Steady state error
EV: Electrical Vehicle
FIS: Fuzzy Inference System
FPIDD^2^: Fuzzy Proportional Integral Derivative Double Derivative Controller
GDB: Dead Band of Governor
GRC: Constraint of Generation Rate (% p.u)
GTO: Gorilla Troops Optimization
ITAE: Integral time absolute error
K_a_: AVR system amplifier gain
K_e_: AVR system exciter gain
K_n_: AVR system generator gain
K_s_: AVR system sensor gain
K_D1_, K_D2_: Derivative gains
K_E_, K_CE_: Fuzzy controller inputs scaling factors
K_EV_: Electric vehicle gain
K_I_: Integral gain
K_P_: Proportional gain
K_ps1_, K_ps2_: Power system gains
K_r_: Steam turbine reheat constant
LFC: Load Frequency Control
M_p-V1_: Maximum overshoot magnitude of terminal voltage of Area (1)
M_p-V2_: Maximum overshoot magnitude of terminal voltage of Area (2)
MO_∆f1_: Maximum Overshoot of Frequency Deviation of Area (1)
MO_∆f2_: Maximum Overshoot of Frequency Deviation of Area (2)
MO_∆P_: Maximum Overshoot of Tie-line Power Deviation
MU_∆f1_: Minimum Overshoot of Frequency Deviation of Area (1)
MU_∆f2_: Minimum Overshoot of Frequency Deviation of Area (2)
MU_∆P_: Minimum Overshoot of Tie-line Power Deviation
MPA: Marine Predator Algorithm
N_1_, N_2_: The coefficients of the filters
PF_hyd_: Hydro unit participation factor
PF_g_: Gas unit participation factor
PF_Th_: Thermal unit participation factor
PF_PV_: PV unit participation factors
PF_WT_: Wind turbine unit participation factor
PID: Proportional Integral Derivative Controller
PIDD^2^: Proportional Integral Derivative Double Derivative Controller
PSO: Particle Swarm Optimization
PV: Photovoltaics
RESs: Sources of renewable energy
R_Th_: Governor speed regulation parameters of thermal unit
R_hyd_: Governor speed regulation parameters of hydro unit
R_g_: Governor speed regulation parameters of gas unit
RLP: Random Load Perturbation
SLP: Step Load Perturbation
T_12_: Synchronizing coefficient
T_a_: AVR system amplifier time constant
T_e_: AVR system exciter time constant
T_n_: AVR system generator time constant
T_s_: AVR system sensor time constant
T_cd_, T_rh_: Compressor discharge volume, and transient droop time constants
T_cr_: The gas turbine combustion reaction’s time delay
T_EV_: Electric vehicle time constant
T_gh_: Hydro turbine governor time constant
T_f_: Gas turbine fuel time constant
T_ps1_, T_ps2_: Time constants of power system
T_r-V1_: Rise time of terminal voltage of Area (1) in seconds
T_r-V2_: Rise time of terminal voltage of Area (2) in seconds
T_rs_: Reset time of speed governor of the Hydro turbine
T_s-V1_: Settling time of terminal voltage of Area (1) in seconds
T_s-V2_: Settling time of terminal voltage of Area (2) in seconds
T_simulation_: Simulation time
T_t_: Time constant of steam turbines
T_sg_: Governor time constant of steam turbine
Tr: Reheat time constant of Steam turbine
Tw: Water starting time in hydro turbine
V_a_: Amplifier output in AVR loop
V_e_: Exciter terminal voltage in AVR loop
V_g_: AVR terminal voltage
V_out1_: Area (1) output terminal voltage (p.u.)
V_out2_: Area (2) output terminal voltage (p.u.)
V_ref_: AVR reference voltage
Vs: Feedback signal in AVR loop
X_g_: The gas turbine governor’s lead time constant
